# Delimitation, new species and teleomorph-anamorph relationships in *Codinaea*, *Dendrophoma*, *Paragaeumannomyces* and *Striatosphaeria* (Chaetosphaeriaceae)

**DOI:** 10.3897/mycokeys.74.57824

**Published:** 2020-10-19

**Authors:** Martina Réblová, Jana Nekvindová, Jacques Fournier, Andrew N. Miller

**Affiliations:** 1 The Czech Academy of Sciences, Institute of Botany, Department of Taxonomy, Průhonice 252 43, Czech Republic Institute of Botany, Czech Academy of Sciences Průhonice Czech Republic; 2 Department of Clinical Biochemistry and Diagnostics, University Hospital Hradec Králové, Hradec Králové 500 05, Czech Republic University Hospital Hradec Králové Hradec Králové Czech Republic; 3 Las Muros, Rimont 09420, France Unaffiliated Rimont France; 4 Illinois Natural History Survey, University of Illinois Urbana-Champaign, Champaign, Illinois 61820, USA University of Illinois Urbana-Champaign Champaign United States of America

**Keywords:** molecular phylogeny, phialidic conidiogenesis, scolecosporous, systematics, wood-inhabiting fungi, 15 new taxa

## Abstract

The Chaetosphaeriaceae are a diverse group of pigmented, predominantly phialidic hyphomycetes comprised of several holomorphic genera including *Chaetosphaeria*, the most prominent genus of the family. Although the morphology of the teleomorphs of the majority of *Chaetosphaeria* is rather uniform, their associated anamorphs primarily exhibit the variability and evolutionary change observed in the genus. An exception from the morphological monotony among *Chaetosphaeria* species is a group characterised by scolecosporous, hyaline to light pink, multiseptate, asymmetrical ascospores and a unique three-layered ascomatal wall. *Paragaeumannomyces sphaerocellularis*, the type species of the genus, exhibits these morphological traits and is compared with similar *Chaetosphaeria* with craspedodidymum- and chloridium-like synanamorphs. Morphological comparison and phylogenetic analyses of the combined ITS-28S sequences of 35 isolates and vouchers with these characteristics revealed a strongly-supported, morphologically well-delimited clade in the Chaetosphaeriaceae containing 16 species. The generic name *Paragaeumannomyces* is applied to this monophyletic clade; eight new combinations and five new species, i.e. *P. abietinus***sp. nov.**, *P. elegans***sp. nov.**, *P. granulatus***sp. nov.**, *P. sabinianus***sp. nov.** and *P. smokiensis***sp. nov.**, are proposed. A key to *Paragaeumannomyces* is provided. Using morphology, cultivation studies and phylogenetic analyses of ITS and 28S rDNA, two additional new species from freshwater and terrestrial habitats, *Codinaea paniculata***sp. nov.** and *Striatosphaeria castanea***sp. nov.**, are described in the family. A codinaea-like anamorph of *S. castanea* forms conidia with setulae at each end in axenic culture; this feature expands the known morphology of *Striatosphaeria*. A chaetosphaeria-like teleomorph is experimentally linked to *Dendrophoma cytisporoides*, a sporodochial hyphomycete and type species of *Dendrophoma*, for the first time.

## Introduction

The family Chaetosphaeriaceae (Réblová et al. 1999) is a speciose, diverse group of pigmented, predominantly phialidic fungi some of which possess known teleomorphs (sexual and asexual morphs, hereafter teleomorph and anamorph respectively). Members of the family have a world-wide geographical distribution. They are essential components of biodiversity and play a role in decomposition of woody and herbaceous material and leaf litter, occur in soil, and some exhibit an endophytic lifestyle and have been isolated from living herbs and trees (e.g. Gams and Holubová-Jechová 1976; Hughes and Kendrick 1968; Réblová and Gams 1999; Réblová and Seifert 2003; Réblová 2004; Fernández and Huhndorf 2005; Huhndorf and Fernández 2005; Crous et al. 2012; Hashimoto et al. 2015; Yang et al. 2018; Lin et al. 2019; Luo et al. 2019).

Sexually reproducing fungi encompassed in the Chaetosphaeriaceae are perithecial ascomycetes that share several morphological traits such as similar anatomy of the brittle, melanised ascomatal wall, persistent paraphyses, unitunicate, thin-walled asci with a refractive, non-amyloid apical annulus, transversely septate ascospores that germinate by germ tubes and phialidic conidiogenesis. Several species produce both ascospores and conidia, ascomata are often associated with conspicuous conidiophores arranged in the juxtaposition. Most representatives of the family reproduce only asexually and are known as “anamorphic holomorphs” (Seifert et al. 2011). They either permanently lost the ability to sexually reproduce and do not develop the teleomorph, or the latter remains to be discovered.

Most of the sexually reproducing fungi in the family were classified in *Chaetosphaeria* (Tulasne & Tulasne, 1863), a prominent genus of the family. *Chloridium botryoideum* has long been known to be a part of the life cycle of *Ch. innumera*, the generic type (Tulasne and Tulasne 1863; Gams and Holubová-Jechová 1976). Using ITS and 28S DNA sequence data, *Ch. innumera* was resolved as unrelated to other *Chaetosphaeria* and chaetosphaeria-like species associated with morphologically different anamorphs (Réblová and Winka 2000; Fernández et al. 2006; Lin et al. 2019). Following the “one fungus, one name” concept (Hawksworth 2011, 2012; Hawksworth et al. 2011), some of the former *Chaetosphaeria* linked with different anamorphs now belong in the respective anamorphic genera based on priority, for example, *Catenularia* (Berkeley and Broome 1871; Hughes 1965a; Holubová-Jechová 1982), *Cacumisporium* (Réblová and Gams 1999), *Chloridium* (Gams and Holubová-Jechová 1976; Réblová et al. 2016), *Exserticlava* (Hino 1961; Matsushima 1985; Réblová and Seifert 2003; Fernández and Huhndorf 2005), *Menispora* (Booth 1957, 1958; Holubová-Jechová 1973; Réblová and Seifert 2008), *Sporoschisma* (Müller et al. 1969, Réblová et al. 2016), *Tainosphaeria* (Fernández and Huhndorf 2005) and *Zanclospora* (Hughes and Kendrick 1965b). Other *Chaetosphaeria* that form natural units, characterised primarily by the morphological traits of their anamorphs, will form the basis of generic classification in the family and, thus, need to be re-examined based on phylogenetic studies.

The majority of species accommodated in *Chaetosphaeria* possess ellipsoidal, fusiform to cylindrical-fusiform, 1–5-septate, hyaline, symmetrical ascospores with their length generally ranging from 6 to 40 μm. Ascomata are brown to black, papillate, often glossy with a two-layered ascomatal wall; the outer layer consisting of several rows of brick-like cells with dark brown, opaque walls. The transfer of a scolecosporous *Lasiosphaeria raciborskii* (Carroll and Munk 1964) with a three-layered ascomatal wall to *Chaetosphaeria* by Miller and Huhndorf (2004) expanded the concept of the genus. Huhndorf and Fernández (2005) introduced another four morphologically similar species based on ITS sequence data, i.e. *Ch. ellisii* (= *Ch. longispora*), *Ch. lapaziana*, *Ch. panamensis* and *Ch. rubicunda*, characterised by unique ascomatal wall anatomy, multiseptate scolecosporous ascospores and occurrence on decaying wood. Their ascomatal wall is composed of three layers. The typical chaetosphaeriaceous outer layer is present as the middle layer, while the outer layer consists of thin-walled, mostly globose cells. The ascospores are hyaline, cylindrical-filiform (up to 150 μm long), 7–16-septate, usually asymmetrical with a bluntly rounded apical end and tapering towards the basal end. These species were experimentally linked with a craspedodidymum-like anamorph, and some also form a chloridium-like synanamorph in axenic culture (Huhndorf and Fernández 2005). Atkinson et al. (2007) and Perera et al. (2016) introduced another three *Chaetosphaeria* matching the diagnostic characters of this group. Among the known ascomycetes, the monotypic genus *Paragaeumannomyces* (Matsushima 2003), based on *P. sphaerocellularis*, is remarkably similar to these scolecosporous species of *Chaetosphaeria* in features of ascomata, asci and ascospores and ecology.

Our sampling of saprobic lignicolous fungi in terrestrial biotopes in various localities in Europe, New Zealand and North America revealed several species whose morphological characters best match those of the genus *Paragaeumannomyces* and other scolecosporous *Chaetosphaeria*, i.e. *Ch. albida* (Atkinson et al. 2007), *Ch. longispora* (Barr 1993; Huhndorf and Fernández 2005) and five unknown species. We also collected additional specimens that represent new species, an unknown *Codinaea* (Maire 1937; Hughes and Kendrick 1968) on submerged wood and leaves in France and United Kingdom and an undescribed *Striatosphaeria* (Samuels and Müller 1978) on decaying bark of a woody liana in French Guiana. *Codinaea*, typified by *C. aristata*, comprises fungi forming tufts of fertile or sterile setae accompanied by conidiophores terminating into a phialide and hyaline, aseptate, falcate conidia with setulae at both ends. *Striatosphaeria* is well distinguishable from other members of the family by brown, 1-septate ascospores with longitudinal ridges and furrows running the entire length of the ascospore and a codinaea-like anamorph with brown, 1-septate conidia.

*Dendrophoma* (Saccardo 1880; Crous et al. 2012) is characterised by superficial, stromatic, stipitate, cupulate conidiomata, phialidic conidiogenous cells arranged in terminal whorls and naviculate to botuliform, aseptate, hyaline conidia with polar appendages. Using DNA sequence data, Crous et al. (2012) confirmed its systematic placement in the Chaetosphaeriaceae. However, its teleomorph-anamorph relationship remains unknown. A collection of a chaetosphaeria-like species with glabrous, dark, erumpent, aggregated ascomata sometimes in caespitose clusters, stipitate asci with inconspicuous apical annulus and fusiform, hyaline, 1-septate ascospores was encountered in the cracks of the bark of twigs of *Buxus sempervivens* in Germany. The axenic culture derived from ascospores yielded an anamorph similar to *Dendrophoma*. A BLASTn search (Zhang et al. 2000) for possible relatives in GenBank (Sayers et al. 2019) suggested our isolate is similar to *Dendrophoma cytisporoides*, the type species of the genus.

The present study provides new data that improve our understanding of morphological and genetic diversity of the Chaetosphaeriaceae and its pleomorphism. Our longer term goals focus on identification of monophyletic, morphologically well-delimited natural lineages and the life history of species currently assigned to the family. To assess phylogenetic relationships of our isolates, we based the study on morphological and cultivation studies along with the analysis of DNA sequence data from the nuc rDNA internal transcribed spacer region (ITS1-5.8S-ITS2 = ITS) and nuclear large subunit 28S ribosomal DNA gene (28S).

## Materials and methods

### Herbarium material and fungal strains

Material for this study was collected in north temperate regions of Europe (France, Germany and Ukraine) and North America (North Carolina, Tennessee), south subtropical and temperate climate zones of New Zealand, and in the neotropical regions of the Caribbean (Puerto Rico) and South America (French Guiana). An additional living culture was obtained from BCCM/MUCL Agro-food & Environmental Fungal Collection (MUCL), Université catholique de Louvain, Louvain, Belgium. Representative strains and ex-type strains were deposited at Westerdijk Fungal Biodiversity Institute (CBS), Utrecht, the Netherlands. Holotypes and other herbarium material (as dried voucher specimens) were deposited in the Fungarium of the Illinois Natural History Survey (ILLS), Champaign, Illinois, USA, the New Zealand Fungarium (PDD), Auckland, New Zealand and Herbarium of the Institute of Botany (PRA), Czech Academy of Sciences, Průhonice, Czech Republic. Isolates and specimens, their sources and GenBank accession numbers for ITS and 28S sequences generated in this study, are listed in Table 1.

**Table 1.** Taxa, isolate information and GenBank accession numbers for new sequences (in bold) determined for this study.

Taxon	Specimen	Status	Country	Host	Substrate	GenBank accessions		Reference
						ITS	28S	
*Codinaea paniculata*	CBS 145098	T	France	unidentified	submerged decaying wood	**MT118230**	**MT118201**	This study
	CBS 126573		France	*Alnus glutinosa*	submerged decaying wood	**MT118231**	**MT118202**	This study
	CBS 127692		France	*Fraxinus excelsior*	submerged decaying wood	**MT118232**	**MT118203**	This study
	MUCL 34876		United Kingdom	unidentified	submerged dead leaf	**MT118233**	**MT118204**	This study
*Dendrophoma cytisporoides*	CBS 144107 IMI 506817		Germany	*Buxus sempervivens*	decaying periderm of a twig	**MT118234**	**MT118205**	This study
*Paragaeumannomyces abietinus*	CBS 145351	T	France	*Abies alba*	decaying wood	**MT118235**	**MT118206**	This study
*Paragaeumannomyces albidus*	PDD 118738		New Zealand	unidentified	decaying wood	**MT876579**	–	This study
*Paragaeumannomyces elegans*	PDD 118740	T	New Zealand	unidentified	decaying wood	**MT876580**	–	This study
*Paragaeumannomyces granulatus*	ICMP 15133	T	New Zealand	unidentified	decaying wood	**MT876575**	**MT876577**	This study
	PDD 118745		New Zealand	unidentified	decaying wood	**MT876576**	**MT876578**	This study
*Paragaeumannomyces lapazianus*	S.M.H. 2182		Costa Rica	unidentified	decaying wood	AY906945	**MT118207**	Huhndorf and Fernández (2005), this study
	S.M.H. 2900		Puerto Rico	unidentified	decaying wood	AY906946	**MT118208**	Huhndorf and Fernández (2005), this study
	S.M.H. 3043		Puerto Rico	unidentified	decaying wood	AY906947	**MT118209**	Huhndorf and Fernández (2005), this study
*Paragaeumannomyces longisporus*	A.N.M. 1269		USA, Tennessee	unidentified	decaying wood	**MT118239**	**MT118210**	This study
	ILLS00121385		USA, Tennessee	unidentified	decaying wood	**MT118237**	**MT118211**	This study
	ILLS00121386		USA, Tennessee	unidentified	decaying wood	**MT118238**	**MT118212**	This study
	S.M.H. 2519		USA, Indiana	unidentified	decaying wood	AY906939	**MT118213**	Huhndorf and Fernández (2005), this study
	S.M.H. 2758		USA, North Carolina	unidentified	decaying wood	AY906940	**MT118214**	Huhndorf and Fernández (2005), this study
	S.M.H. 3805		USA, North Carolina	unidentified	decaying wood	**MT118236**	**MT118215**	This study
	S.M.H. 3809		USA, North Carolina	unidentified	decaying wood	AY906942	**MT118216**	Huhndorf and Fernández (2005), this study
	S.M.H. 3860		USA, South Carolina	unidentified	decaying wood	AY906944	**MT118217**	Huhndorf and Fernández (2005), this study
*Paragaeumannomyces panamensis*	S.M.H. 3596	T	Panama	unidentified	decaying wood	AY906948	**MT118218**	Huhndorf and Fernández (2005), this study
*Paragaeumannomyces* sp. 1	S.M.H. 2025		Puerto Rico	unidentified	decaying wood	**MT118241**	**MT118219**	This study
	S.M.H. 3014		Puerto Rico	unidentified	decaying wood	AY906952	**MT118222**	Huhndorf and Fernández (2005) (as ‘*raciborskii*’), this study
*Paragaeumannomyces* sp. 2	S.M.H. 2036		Puerto Rico	unidentified	decaying wood	AY906950	**MT118220**	Huhndorf and Fernández (2005) (as ‘*raciborskii*’), this study
	S.M.H. 2132		Puerto Rico	unidentified	decaying wood	AY906951	**MT118221**	Huhndorf and Fernández (2005) (as ‘*raciborskii*’), this study
*Paragaeumannomyces rubicundus*	S.M.H. 2881	PT	Puerto Rico	unidentified	decaying wood	AY906954	**MT118223**	Huhndorf and Fernández (2005), this study
	S.M.H. 3221	T	Costa Rica	unidentified	decaying wood	**MT118242**	**MT118224**	This study
*Paragaeumannomyces sabinianus*	ILLS00121384	T	USA, Tennessee	unidentified	decaying wood	**MT118243**	**MT118225**	This study
	S.M.H. 3807		USA, North Carolina	unidentified	decaying wood	AY906941	**MT118226**	Huhndorf and Fernández (2005), this study
	S.M.H. 3824		USA, North Carolina	unidentified	decaying wood	AY906943	**MT118227**	Huhndorf and Fernández (2005), this study
*Paragaeumannomyces smokiensis*	ILLS00121398	T	USA, Tennessee	unidentified	decaying wood	**MT118240**	**MT118228**	This study
*Striatosphaeria castanea*	CBS 145352	T	French Guinea	woody liana	decaying periderm	**MT118244**	**MT118229**	This study
*Striatosphaeria codinaeophora*	S.M.H. 1524		Puerto Rico	*Nectandra turbacensis*	decaying wood	**MT118245**	AF466088	Huhndorf et al. (2004), this study

Note: T and PT denote ex-type and ex-paratype strains.

### Morphological characterisation

Morphological characteristics were obtained from fungi growing on natural substrate and growth media. Descriptions in the key are based on fungi growing on natural substrate. Herbarium material was rehydrated with tap water and examined with an Olympus SZX12 dissecting microscope (Olympus America, Inc., Melville, USA). Hand-sectioned ascomata and centrum material (asci, ascospores and paraphyses), conidiophores and conidia were mounted in 90 % lactic acid, Melzer’s reagent, and lactophenol with cotton blue. All measurements were in Melzer’s reagent. Means ± standard deviation (SD) based on a minimum of 20–25 measurements are given for dimensions of asci, ascospores and conidia. Micromorphological observations were made using an Olympus BX51 compound microscope with differential interference contrast (DIC) and phase contrast (PC) illumination. Images of microscopic structures were captured with an Olympus DP70 camera operated by Imaging Software Cell^D (Olympus). Macroscopic images of colonies were documented using a Canon EOS 77D digital camera with Canon EF 100mm f/2.8L Macro IS USM objective with daylight spectrum 5500K 16W LED lights (Canon Europe Ltd., Middlesex, United Kingdom). All images were processed with Adobe Photoshop CS6 (Adobe Systems, San Jose, USA).

For comparative purposes, strains were inoculated in triplicate on cornmeal dextrose agar (CMD) [17 g of cornmeal agar (Oxoid Limited, Hampshire, United Kingdom), 2 g of dextrose, 1 L of distilled water, sterilized for 15 min at 121 C], Modified Leonian’s agar (MLA) (Malloch 1981), oat-meal agar (OA) modified after Gooding and Lucas (1959) (30 g of oatmeal cooked in 1 L of distilled water for 15–30 min, filtered through cheesecloth, the filtrate was brought back to volume with distilled water, 15 g of agar, sterilized for 60 min at 121 C) and potato-carrot agar (PCA) (Crous et al. 2019). To induce sporulation, strains were also inoculated on CMA (Crous et al. 2019) with sterile stems of *Urtica dioica*. Descriptions of colonies are based on 4 wk old cultures grown in darkness at 22–23 C.

### DNA extraction and amplification

Methods for the DNA extraction and amplification of samples with A.N.M., ILLS and S.M.H. prefixes followed Huhndorf et al. (2004) and Hustad and Miller (2015). Other samples were processed according to the following protocols. Total genomic DNA was extracted from mycelium removed from 3-wk-old cultures grown on MLA using the DNeasy UltraClean Microbial Kit (Qiagen GmbH, Germany) following the manufacturer’s protocol for filamentous fungi. All PCR amplifications were carried out in 25 μL volume reactions using a Q5 High Fidelity DNA polymerase kit (New England Biolabs Inc., United Kingdom) according to the manufacturer’s protocol. Primers used for the amplification included: V9G/LR8 (de Hoog and Gerrits van den Ende 1998; Vilgalys unpublished) for the internal transcribed spacers (ITS) of the nuclear rRNA cistron and D1, D2 and D3 domains (approx. 1900 bp of the 5' end) of the 28S rDNA gene.

PCR was carried out in a BioRad C1000 thermal cycler (Bio-Rad Laboratories Inc., USA) as follows: 98 C for 30 s; 40 cycles of denaturation (98 C for 10 s), annealing (62 C for 30 s) and elongation (72 C for 90 s) and a final extension step at 72 C for 5 min. Amplicons were purified from agarose gels using a NucleoSpin® Gel and PCR Clean-up Kit (Macherey-Nagel GmbH & Co. KG, Germany) following the manufacturer’s instructions, with an elution volume of 25 μL. The DNA concentration was assessed fluorimetrically using Quant-iT PicoGreen dsDNA Assay Kit and Qubit fluorometer (Invitrogen / Thermo Fisher Scientific, USA) to assure required sequencing concentrations adjusted for the length of amplicons/ number of reads required.

Each of the amplicons was sequenced in both directions using the PCR primers and nested primers: ITS5, ITS4, JS1, JS7, JS8 and LR7 for ITS-28S (Vilgalys and Hester 1990; White et al. 1990; Landvik 1996; Vilgalys unpublished). Automated sequencing was carried out by Eurofins GATC Biotech Sequencing Service (Cologne, Germany). Raw sequence data were assembled, examined and edited using Sequencher v.5.4.6 (Gene Codes Corp., Ann Arbor, USA).

### Alignments and phylogenetic analyses

Two gene markers, ITS and 28S rDNA, were analysed to assess evolutionary relationships of the unknown fungi with members of the Chaetosphaeriaceae. Consensus secondary structure (2D) models for the ITS1 and ITS2 for members of the Chaetosphaeriaceae were built using the Ppfold program v.3.0 (Sukosd et al. 2012). The obtained 2D consensus models were further improved using the program Mfold (Zuker 2003) and adjusted manually if necessary, based on comparison of homologous positions in the multiple sequence alignment. A predicted 2D model of the 28S of *Saccharomyces cerevisiae* (Gutell et al. 1993) was used to improve the alignment of this gene. The models were highly consistent in all taxa.

ITS and 28S sequences were aligned manually in Bioedit v.7.1.8 (Hall 1999). GenBank accession numbers for ITS and 28S sequences of members of the Chaetosphaeriaceae retrieved from GenBank and published in other studies are listed in Table 2. Single-locus data sets of the Chaetosphaeriaceae (ITS: 89 sequences/602 characters including gaps, 28S: 86/1176) and *Paragaeumannomyces* (ITS: 35/489, 28S: 32/1104) were evaluated using PartitionFinder2 (Lanfear et al. 2016), implemented in the CIPRES Science Gateway v.3.3 (http://www.phylo.org) (Miller et al. 2010), to find the best partitioning scheme for our datasets and to select best-fit models under corrected Akaike information criteria. Conflict-free data sets were concatenated into two alignments (deposited in TreeBASE 25964) that were subjected to subsequent phylogenetic analyses.

Three analyses were employed to estimate phylogenetic relationships. Bayesian Inference (BI) and Maximum Likelihood (ML) analyses were performed through the CIPRES Science Gateway v.3.3. ML analyses were conducted with RAXML-HPC v.8.2.12 (Stamatakis 2014) with a GTRCAT approximation. Nodal support was determined by non-parametric bootstrapping (BS) with 1 000 replicates. BI analyses were performed in a likelihood framework as implemented in MrBayes v.3.2.6 (Huelsenbeck and Ronquist 2001). Two Bayesian searches were performed using default parameters. The B-MCMCMC analyses lasted until the average standard deviation of split frequencies was below 0.01 with trees saved every 1000 generations. The first 25 % of saved trees, representing the burn-in phase of the analysis, were discarded. The remaining trees were used for calculating posterior probabilities (PP) of recovered branches. Maximum Parsimony (MP) analyses were conducted with PAUP 4.0a167 (Swofford 2003). A heuristic search was performed with the stepwise-addition option with 1000 random taxon addition replicates and TBR branch swapping. Because the secondary structures of the ITS and 28S were carefully studied when aligning the sequences, and regions with incomplete sequences were excluded from the analysis, we treated gaps as a fifth character state; they were given equal weight as the other characters. All characters were unordered. Branch support was estimated on the recovered topologies by performing a heuristic search of 1000 bootstrap replicates consisting of ten random-addition replicates for each bootstrap replicate.

**Table 2.** Taxa, isolate information and accession numbers for sequences retrieved from GenBank.

Taxon	Strain	Status	Country	Host	Substrate	GenBank accessions		Reference
						ITS	28S	
*Adautomilanezia caesalpiniae*	CC-LAMIC 102/12	T	Brazil	*Caesalpina echinata*	wood	KX821777	KU170671	Crous et al. (2016)
*Anacacumisporium appendiculatum*	HMAS 245593	T	China, Hainan	broad-leaved tree	dead stems	KP347129	KT001553	Ma et al. (2016)
*Brunneodinemasporium brasiliense*	CBS 112007	T	Brazil	unidentified	decaying leaf	JQ889272	JQ889288	Crous et al. (2012)
*Brunneodinemasporium jonesii*	GZCC 16-0050	T	China	unidentified	decaying wood	KY026058	KY026055	Lu et al. (2016)
*Cacumisporium capitulatum*	FMR 11339		Spain	unidentified	decaying wood	HF677176	HF677190	Hernández-Restrepo et al. (2017)
*Calvolachnella guaviyunis*	CBS 134695	T	Uruguay	*Myrcianthes pungens*	bark	KJ834524	KJ834525	Crous et al. (2014a)
*Chaetosphaeria chlorotunicata*	S.M.H. 1565	T	Puerto Rico	unidentified	decaying wood	–	AF466064	Fernández et al. (2006)
*Chaetosphaeria innumera*	M.R. 1175		Czech Republic	*Fagus sylvatica*	decaying wood	AF178551	AF178551	Réblová and Winka (2000)
*Chaetosphaeria lignomollis*	S.M.H. 3015	T	Puerto Rico	unidentified	decaying wood	EU037896	AF466073	Atkinson et al. (2007), Fernández et al. (2006)
*Chaetosphaeria myriocarpa*	CBS 264.76		The Netherlands	unidentified	decaying wood	AF178552	AF178552	Réblová and Winka (2000)
*Chaetosphaeria pygmaea*	M.R. 1365		Czech Republic	*Fagus sylvatica*	decaying wood	AF178545	AF178545	Réblová and Winka (2000)
*Chloridium caesium*	CBS 102339		Austria	*Salix cinerea*	decaying wood	AF178564	AF178564	Réblová and Winka (2000)
*Chloridium gonytrichii*	CBS 195.60		South Africa	unidentified	unknown	MH857954	MH869503	Vu et al. (2019)
*Chloridium virescens*	CBS 152.53		France	*Acer* sp.	unknown	MH857142	MH868678	Vu et al. (2019)
*Codinaea acaciae*	CBS 139907	T	Malaysia, Sarawak	*Acacia mangium*	leaf spot	KR476732	–	Crous et al. (2015b)
*Codinaea lambertiae*	CBS 143419	T	Australia, N.S. Wales	*Lambertia formosa*	leaves	MG386052	MG386105	Crous et al. (2017)
*Codinaea pini*	CBS 138866	T	Uganda	*Pinus patula*	dead needles	KP004465	KP004493	Crous et al. (2014b)
*Codinaea simplex*	CBS 966.69		The Netherlands	*Quercus* sp.	cupule	AF178559	AF178559	Réblová and Winka (2000)
*Codinaeopsis gonytrichodes*	CBS 593.93		Japan	unidentified	decaying plant material	AF178556	AF178556	Réblová and Winka (2000)
*Conicomyces pseudotransvaalensis*	HHUF 29956	T	Japan	*Machilus japonica*	dead twig	LC001710	LC001708	Liu et al. (2015)
*Cryptophiale udagawae*	GZCC 18-0047		China, Guizhou	unidentified	decaying wood	MN104608	MN104619	Lin et al. (2019)
*Dendrophoma cytisporoides*	CBS 223.95	ET	The Netherlands	*Rhododendron* sp.	branches and twigs	JQ889273	JQ889289	Crous et al. (2012)
*Dictyochaeta assamica*	CBS 242.66		Guadeloupe	*Musa* sp.	root	MH858788	MH870426	Vu et al. (2019)
*Dictyochaeta callimorpha*	ICMP 15130		New Zealand	unidentified	decaying wood	MT454483	MT454498	Réblová et al. (2020)
*Dictyochaeta cangshanensis*	MFLUCC 17-2214	T	China, Yunnan	unidentified	submerged decaying wood	MK828632	MK835832	Luo et al. (2019)
*Dictyochaeta ellipsoidea*	MFLUCC 18-1574	T	China, Yunnan	unidentified	submerged decaying wood	MK828628	MK835828	Luo et al. (2019)
*Dictyochaeta fuegiana*	ICMP 15153	T	New Zealand	unidentified	decaying wood	MT454487	EF063574	Réblová and Seifert (2007), Réblová et al. (2020)
*Dictyochaeta lignicola*	DLUCC 0899	T	China, Yunnan	unidentified	submerged decaying wood	MK828630	MK835830	Luo et al. (2019)
*Dictyochaeta pandanicola*	KUMCC 16-0153	T	China, Yunnan	*Pandanus* sp.	decayng leaf	MH388338	MH376710	Tibpromma et al. (2018)
*Dictyochaeta septata*	CBS 143386	ET	Chile	Eucalyptus grandis × urophylla	leaves	MH107889	MH107936	Crous et al. (2018a)
*Dictyochaeta siamensis*	MFLUCC 15-0614	T	Thailand	unidentified	submerged decaying twig	KX609955	KX609952	Liu et al. (2016)
*Dictyochaeta terminalis*	GZCC 18-0085	T	China, Guizhou	unidentified	decaying leaves	MN104613	MN104624	Lin et al. (2019)
*Dinemasporium americanum*	CBS 127127	T	USA, Iowa	n/a	soil of tallgrass prairie	JQ889274	JQ889290	Crous et al. (2012)
*Dinemasporium pseudoindicum*	CBS 127402	T	USA, Kansas	n/a	soil of tallgrass prairie	JQ889277	JQ889293	Crous et al. (2012)
*Ellisembia aurea*	CBS 144403	T	France	*Sambucus nigra*	decaying wood	MH836375	MH836376	Hyde et al. (2019)
*Ellisembia folliculata*	CBS 101317		France	*Salix* sp.	decaying wood	–	AF261071	Réblová and Winka (2001)
*Eucalyptostroma eucalypti*	CBS 142074	T	Malaysia	*Eucalyptus pellita*	leaf spots	KY173408	KY173500	Crous et al. (2016)
*Exserticlava vasiformis*	TAMA 450		Japan, Chiba	unidentified	plant debris	–	AB753846	Tsuchiya et al., unpublished
*Infundibulomyces cupulatus*	BCC 11929	T	Thailand	*Lagerstroemia* sp.	dead leaf	EF113976	EF113979	Plaingam et al. (2003)
*Infundibulomyces oblongisporus*	BCC 13400	T	Thailand	unidentified, angiosperm	leaf litter	EF113977	EF113980	Somrithipol et al. (2008)
*Kionochaeta castaneae*	GZCC 18-0025	T	China	*Castanea mollissima*	decaying seed shell	MN104610	MN104621	Lin et al. (2019)
*Kionochaeta microspora*	GZCC 18-0036	T	China, Guizhou	unidentified	decaying wood	MN104607	MN104618	Lin et al. (2019)
*Leptosporella arengae*	MFLUCC 15-0330	T	Thailand	*Arenga pinnata*	dead rachis	MG272255	MG272246	Konta et al. (2017)
*Leptosporella bambusae*	MFLUCC 12-0846	T	Thailand	bamboo	dead culms	KU940134	KU863122	Dai et al. (2016)
*Menispora ciliata*	CBS 122131	T	Czech Republic	*Acer campestre*	decaying wood	EU488736	–	Réblová and Seifert (2008)
*Menispora tortuosa*	DAOM 231154		unknown	unidentified	unknown	KT225527	AY544682	Schoch et al. (2009)
*Menisporopsis dushanensis*	GZCC 18-0084	T	China, Guizhou	unidentified	decaying leaves	MN104615	MN104626	Lin et al. (2019)
*Menisporopsis theobromae*	MFLUCC 15-0055		Thailand	unidentified	submerged decaying wood	KX609957	KX609954	Liu et al. (2016)
*Nawawia filiformis*	MFLUCC 17-2394		Thailand	unidentified	decaying wood	MH758196	MH758209	Yang et al. (2018)
*Neopseudolachnella acutispora*	MAFF 244358	T	Japan, Aomori	*Pleioblastus chino*	dead twigs	AB934065	AB934041	Hashimoto et al. (2015)
*Neopseudolachnella magnispora*	MAFF 244359	T	Japan, Aomori	*Sasa kurilensis*	dead twigs	AB934066	AB934042	Hashimoto et al. (2015)
*Paliphora intermedia*	CBS 896.97	IST	Australia, Queensland	unidentified	leaf litter	MH862682	EF204501	Shenoy et al. (2010), Vu et al. (2019)
*Paragaeumannomyces albidus*	PDD 92537	T	New Zealand	*Nothofagus* sp.	decaying wood	EU037890	EU037898	Atkinson et al. (2007)
*P. albidus*	PDD 92540		New Zealand	*Nothofagus* sp.	decaying wood	EU037891	–	Atkinson et al. (2007)
*Paragaeumannomyces bombycinus*	PDD 92538	T	New Zealand	*Nothofagus* sp.	decaying wood	EU037892	–	Atkinson et al. (2007)
*Paragaeumannomyces elegans*	PDD 92561		New Zealand	unidentified	decaying wood	EU037895	–	Atkinson et al. (2007)
*Paragaeumannomyces garethjonesii*	MFLUCC 15-1012	T	Thailand	Fabaceae	seed pod	KY212751	KY212759	Perera et al. (2016)
*Paragaeumannomyces panamensis*	MFLUCC 15-1011		Thailand	*Pinus* sp.	decaying wood	KY212752	KY212760	Perera et al. (2016)
*Paragaeumannomyces* sp. 3	S.M.H. 2017		Puerto Rico	unidentified	decaying wood	AY906949	AF466078	Huhndorf and Fernández (2005) (as ‘*raciborskii*’)
*Paragaeumannomyces* sp. 4	S.M.H. 3119		Puerto Rico	unidentified	decaying wood	AY906953	AY436402	Huhndorf and Fernández (2005) (as ‘*raciborskii*’)
*Phialosporostilbe scutiformis*	MFLUCC 17-0227	T	China	unidentified	submerged decaying wood	MH758194	MH758207	Yang et al. (2018)
*Polynema podocarpi*	CBS 144415	T	New Zealand	*Podocarpus totara*	unknown	MH327797	MH327833	Crous et al. (2018b)
*Pseudodinemasporium fabiforme*	CBS 140010		Malaysia, Sarawak	*Acacia mangium*	leaf spots	KR611889	KR611906	Crous et al. (2015a)
*Pseudolachnea fraxini*	CBS 113701	T	Sweden	*Fraxinus excelsior*	unknown	JQ889287	JQ889301	Crous et al. (2012)
*Pseudolachnea hispidula*	MAFF 244365		Japan, Aomori	*Morus bombycis*	dead twig	AB934072	AB934048	Hashimoto et al. (2015)
*Pseudolachnella asymmetrica*	MAFF 244366		Japan, Fukuoka	Phyllostachys nigra var. henonis	dead twig	AB934073	AB934049	Hashimoto et al. (2015)
*Pseudolachnella scolecospora*	MAFF 244379		Japan, Gifu	Sasa sp.	dead twigs	AB934086	AB934062	Hashimoto et al. (2015)
*Pyrigemmula aurantiaca*	CBS 126743	T	Hungary	*Vitis vinifera*	bark	HM241692	HM241692	Magyar et al. 2011)
*Sporoschisma longicatenatum*	MFLUCC 16-0180	T	Thailand	unidentified	submerged decaying wood	KX505871	KX358077	Yang et al. (2016)
*Sporoschisma mirabile*	FMR 11247		Spain	unidentified	dead wood	HF677174	HF677183	Hernández-Restrepo et al. (2017)
*Striatosphaeria castanea*	monte6.2		Brazil	*Encyclia ghillanyi*	root	KC928368	–	Almeida et al., unpublished
*Striatosphaeria codinaeophora*	M.R. 1230		Puerto Rico	*Dacryodes excelsa*	decaying wood	AF178546	AF178546	Réblová and Winka (2000)
*Tainosphaeria jonesii*	GZCC 16-0065	PT	China, Guangxi	unidentified	submerged decaying wood	KY026060	KY026057	Lu et al. (2016)
*Tainosphaeria siamensis*	MFLUCC 15-0607	T	Thailand	unidentified	submerged decaying wood	KX609956	KX609953	Liu et al. (2016)
*Thozetella nivea*	n/a		unknown	unidentified	unknown	EU825201	EU825200	Jeewon et al. (2009)
*Thozetella tocklaiensis*	CBS 378.58	T	India	*Camellia sinensis*	decaying flower	MH857817	MH869349	Vu et al. (2019)
*Tracylla aristata*	CBS 141404	ET	Australia, Victoria	*Eucalyptus regnans*	leaf	KX306770	KX306795	Hernández-Restrepo et al. (2016)
*Tracylla eucalypti*	CBS 144429	T	Colombia	*Eucalyptus urophylla*	spots on living leaves	MH327810	MH327846	Crous et al. (2018b)
*Zanclospora iberica*	CBS 130426	T	Spain	unidentified	decaying wood	KY853480	KY853544	Hernández-Restrepo et al. (2017)

Note: T, ET, IST and PT denote ex-type, ex-epitype, ex-isotype and ex-paratype strains.

## Results

### Phylogenetic analyses

Employing the predicted 2D structure of the variable ITS region and 28S enabled us to construct a reliable multiple sequence alignment of homologous positions at both helices and loops, thus eliminating potential ambiguous regions in the alignments. Initially, we compared trees from ML phylogenetic analyses of the two combined data sets (Chaetosphaeriaceae and scolecosporous species of *Chaetosphaeria*) after alignments were improved with the 2D structure, with and without applying Gblocks (Castresana 2000) using default options, to delimit and remove putative ambiguous regions. The phylogenetic trees based on datasets using Gblocks had lower support for nodes and relationships within and among several clades that could not be resolved (data not shown) compared to trees in which these regions remained. Therefore, the final phylogenies were based on datasets in which Gblocks was not employed.

**Figure 1.**
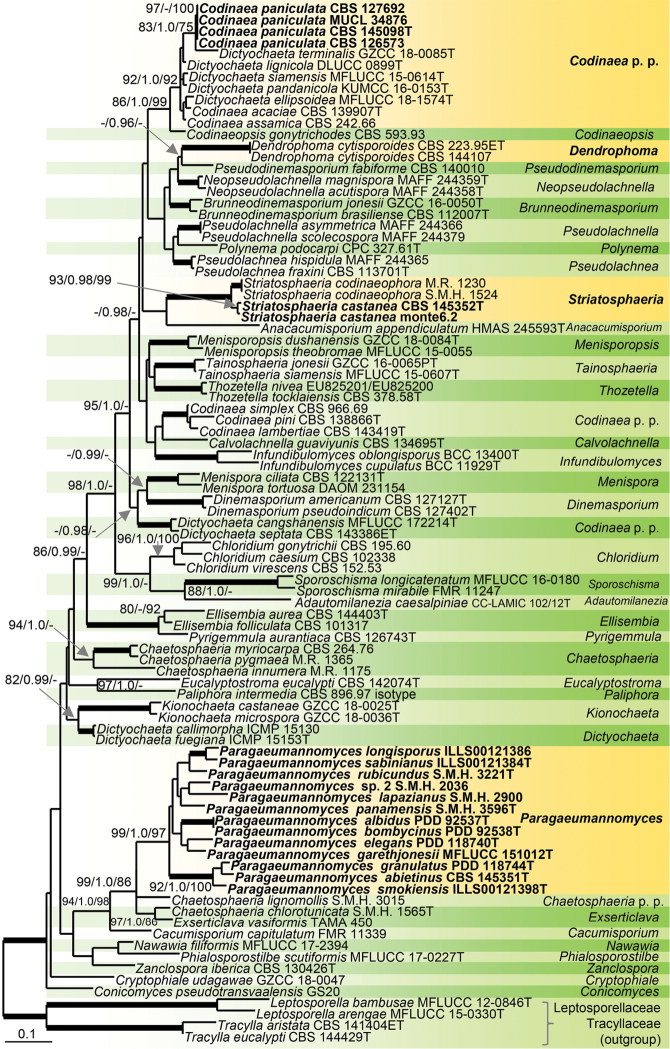
Combined phylogeny using ITS and 28S of selected members of the Chaetosphaeriaceae. Species names given in bold are taxonomic novelties; T, ET, IST and PT indicate ex-type, ex-epitype, ex-isotype and ex-paratype strains. Thickened branches indicate branch support with MLBS = 100%, PP values = 1.0 and MP = 100 %. Branch support of nodes ≥ 75 % ML and MPBS, and ≥ 0.95 PP is indicated above branches.

Evolutionary relationships of studied fungi were evaluated in the phylogenetic analysis based on the combined ITS and 28S sequences of 87 representative species of the Chaetosphaeriaceae. *Leptosporella arengae*, *L. bambusae* (Leptosporellaceae), and *Tracylla eucalypti* and *T. aristata* (Tracyllaceae) were used to root the tree. 76 nucleotides (nt) at the 5'-end and 606 nt at the 3'-end of 28S were excluded from the alignment because of missing data in the majority of sequences. The alignment had 1778 characters including gaps and 882 unique character sites (RAxML). In the MP analysis, 1021 characters were constant (proportion = 57.42 %), 134 variable characters were parsimony-uninformative, 623 characters were parsimony-informative (included); two most parsimonious trees were produced (length = 5066 steps, consistency index = 0.0.298, homoplasy index = 0.702, retention index = 0.631). For the BI analysis, GTR+I+G model was selected for ITS and 28S partitions. The ML tree (RAxML) is shown in Fig. 1. There were no conflicts among the trees generated by the three different phylogenetic analyses. The Chaetosphaeriaceae were resolved as a strongly supported clade; some of the nodes of the backbone tree, which obtained support in the ML and/or BI analyses, were not statistically supported in MP analysis. The 39 identified terminal clades corresponded to individual genera or natural groups of species. *Codinaea* was resolved as polyphyletic in three subclades. The unknown species was grouped in a clade (92 % MLBS/1.0 PP/92 % MPBS) containing seven *Codinaea* or *Dictyochaeta* species, three of which possess the typical *Codinaea* phenotype, while other morphologically similar species with setulate conidia clustered in the other two subclades, *C. lambertiae*, *C. pini* and *C. simplex* (100/1.0/100) and *Dictyochaeta septata* and *D. cangshanensis* (96/1.0/100). The new species *Codinaea paniculata*, based on four strains, was resolved as a monophyletic clade in all three analyses, although the statistical support varied. In ML and MP analyses the clade obtained 97 % and 100 % support, respectively, in the BI analysis it was weakly supported with 0.77 PP. The intraspecific variability of *C. paniculata*, based on ITS sequences, varied slightly. Three strains (CBS 145098 ex-type, CBS 126573, MUCL 34876) had identical ITS sequences, strain CBS 127692 differed from them by one base pair. The 28S sequence similarity of all strains of *C. paniculata* was 100 %. The undescribed *Striatosphaeria* was nested in the monophyletic *Striatosphaeria* (100/1.0/100) clade as sister to *S. codinaeophora*. It clustered in a subclade (93/0.98/99) with an endophytic isolate *Striatosphaeria* sp. monte6.2; their ITS exhibited 98.5 % sequence similarity. The five unknown chaetosphaeria-like species with scolecosporous ascospores were nested in a strongly-supported monophyletic clade (99/1.0/97). This clade contained eight additional morphologically similar species with scolecosporous ascospores and three-layered ascomatal wall. The clade is introduced as *Paragaeumannomyces* in this study. A chaetosphaeria-like species grouped with the ex-type strain of *Dendrophoma cytisporoides*CBS 223.95 in a monophyletic clade (100/1.0/100). *Dendrophoma* was resolved as a member of a large, statistically weakly-supported grouping containing six other genera characterised by sporodochial conidiomata.

**Figure 2.**
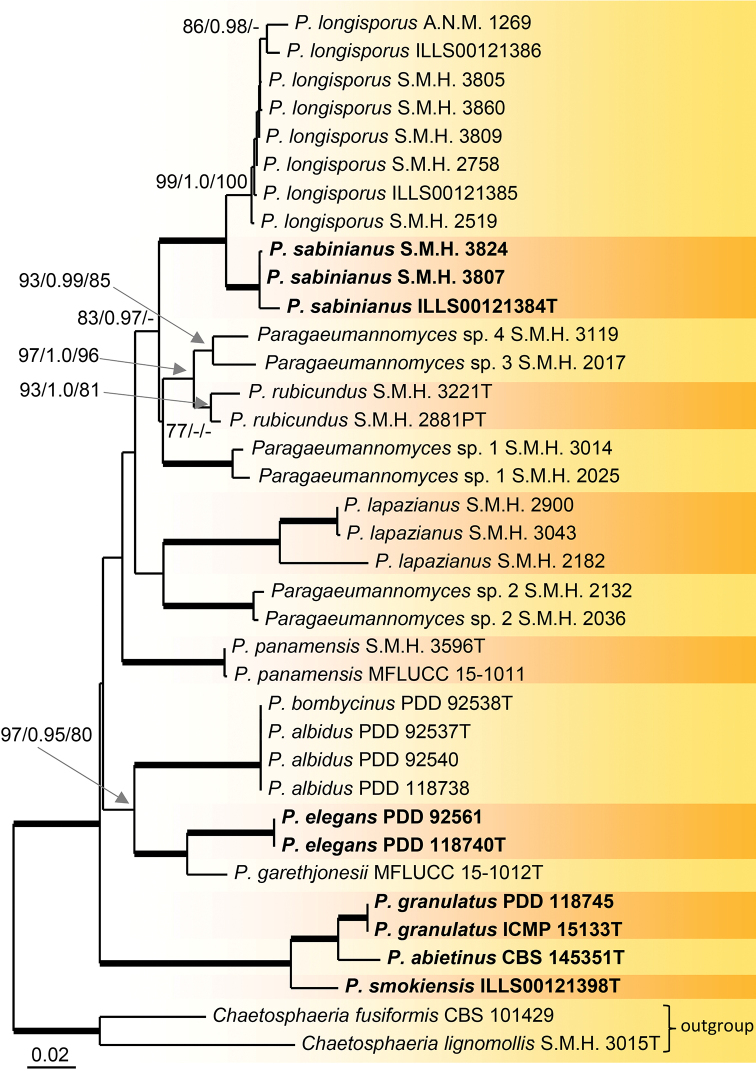
Combined phylogeny using ITS and 28S of 35 members of *Paragaeumannomyces*. Species names given in bold are new species; T and PT indicate ex-type and ex-paratype strains. Thickened branches indicate branch support with MLBS = 100%, PP values = 1.0 and MP = 100 %. Branch support of nodes ≥ 75 % ML and MPBS, and ≥ 0.95 PP is indicated above branches.

Phylogenetic relationships within the genus *Paragaeumannomyces* were assessed in the second analysis of the combined ITS-28S loci. *Chaetosphaeria fusiformis* and *Ch. lignomollis* (Chaetosphaeriaceae) were used to root the tree, and thus served as outgroup. The analysis included 35 sequences belonging to 16 species. 28 nt at the 5'-end and 714 nt at the 3'-end of 28S were excluded from the alignment due to missing data in the majority of sequences. The alignment had 1593 characters including gaps and 410 unique character sites (RAxML). In the MP analysis, 1247 characters were constant (proportion = 78.28 %), 78 variable characters were parsimony-uninformative, and 268 characters were parsimony-informative (included); 286 most parsimonious trees were produced (length = 832 steps, consistency index = 0.6118, homoplasy index = 0.3882, retention index = 0.8082). For the BI analysis, SYM+G and GTR+I+G models were selected for ITS and 28S partitions, respectively. The ML tree is shown in Fig. 2. There were no conflicts among the trees generated by the three different phylogenetic analyses. In the MP strict consensus tree, branches collapsed within the *P. longisporus*, *P. sabinianus* and *P. albidus-bombycinus* clades. The unknown species from wood of *Abies alba* clustered in a subclade (100/1.0/100) with two other unknown species from New Zealand and USA. They were introduced as new species sharing similar ascoma morphology, i.e. *P. abietinus*, *P. granulatus* and *P. smokiensis*. Another unknown species from New Zealand with densely setose, brownish-grey ascomata was grouped as a sister to *P. garethjonesii* and is introduced as *P. elegans*. The subclade (100/1.0/100) identified initially as *Chaetosphaeria ellisiifide*Huhndorf and Fernández (2005) [= *Chaetosphaeria longisporafide*Kirk (2014)] was segregated into two well-supported subclades distinguished by ascospore morphology. These subclades represent two species, *P. longisporus* (99/1.0/100) and the new species, *P. sabinianus* (100/1.0/100). *Paragaeumannomyces raciborskiifide*Huhndorf and Fernández (2005) was resolved as polyphyletic forming four subclades accompanied by different anamorph morphology. Because none of these subclades could be designated ‘*raciborskii* s. str.’, they were labelled *Paragaeumannomyces* sp. 1–4.

### Taxonomy

#### Codinaea paniculata

Taxon classificationFungiChaetosphaerialesChaetosphaeriaceae

Réblová & J. Fourn. sp. nov.

900DCFCB-3172-5D7C-9453-8DE8414B9B09

836526

Figure 3

##### Typification.

France – Ariège • Pyrénées Mts., Rimont, La Maille brook; alt. 550 m; 28 May 2018 (incubated in moist chamber for 1 wk); on submerged decaying wood; J. Fournier leg.; M.R. 3950 (**holotype**: PRA-16319!, ex-type culture CBS 145098).

##### Etymology.

*Panicula* (Latin) tuft, referring to the dense groups of setae and conidiophores on the natural substrate.

##### Description on the natural substrate.

Colonies on the nature substrate effuse, hairy, greyish-brown. Setae erect, straight or slightly flexuous, smooth-walled, dark brown and thick-walled, becoming pale brown to subhyaline and thin-walled towards the apex, 230–290 μm long, 6–7.5 μm wide above the base, tapering gradually towards the apex which almost always develops into a monophialide. Conidiophores macronematous, mononematous, 62–127 × 3.5–4.5 µm, septate, erect, straight or flexuous, arising singly or in groups of 4–6 from hyphal cells associated with the bases of setae, septate, mid-brown to pale brown becoming gradually paler towards the apex. Conidiogenous cells 16.5–30(–38) × 3.5–5 μm, tapering to 1.5–2 μm just below the collarette, integrated, terminal, monophialidic, cylindrical to cylindrical-lageniform, subhyaline or pale brown at the base becoming hyaline to subhyaline towards the apex, smooth-walled; collarettes funnel-shaped, 3.5–4.5 μm wide, 1.5–2.5 μm deep. Conidia in slimy droplets, hyaline in mass, (11.5–)12–17 × (2–)2.5–3(–3.5) µm (mean ± SD = 14.7 ± 1.5 × 2.5 ± 0.3 µm), of two types, narrower and longer, 13.5–17(–17.5) × 2.5–3.5 μm (mean ± SD = 15.2 ± 1.0 × 2.8 ± 0.3 µm), and shorter and usually wider, 11.5–13.5(–14) × 3–3.5(–4) μm, falcate, asymmetrical, rounded at the apical end, with an inconspicuous scar at the basal end, hyaline, aseptate, smooth-walled, with simple, straight or gently curved setulae at both ends, 5–8 μm long; setulae inserted on the concave sides of the conidia.

**Figure 3.**
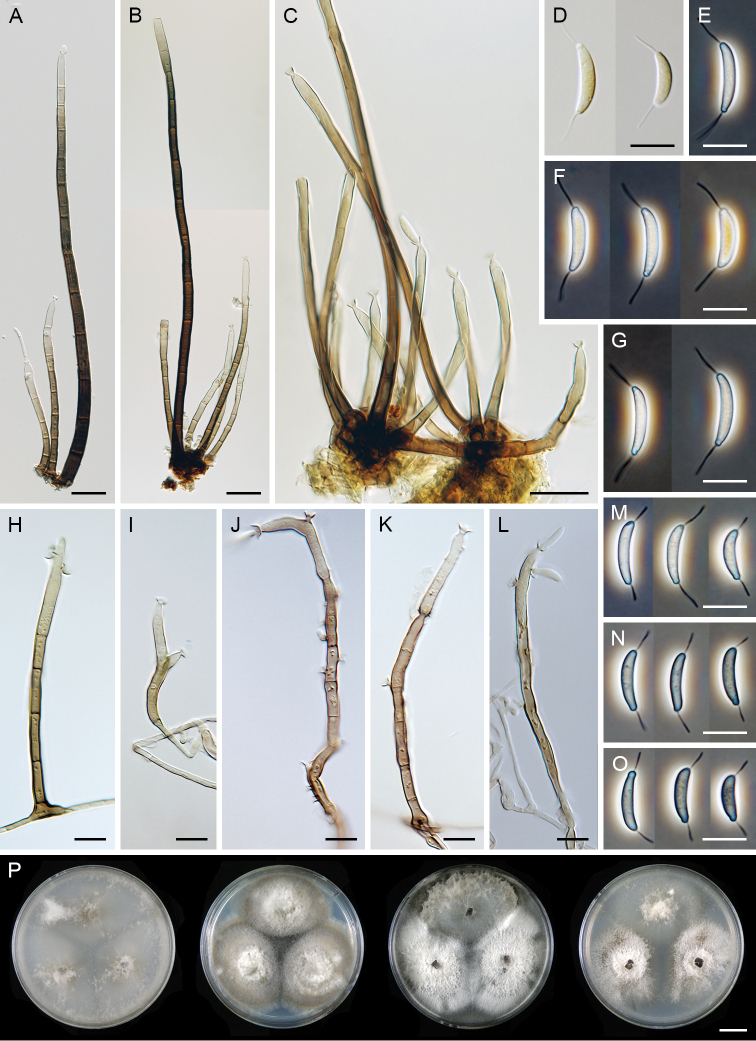
*Codinaea paniculata*. **A–C** setae and conidiophores on nature substrate **D–G** conidia on nature substrate **H–L** conidiophores in MLA culture (6 wk) **M–O** conidia in MLA culture (6 wk) **P** colonies on CMD, MLA, OA and PCA after 4 wk (from left to right). Images: CBS 145098 (**A, B, G–O**); CBS 126573 (**C**); CBS 127692 (**D–F**). Scale bars: 20 μm (**A–C**); 10 μm (**D–O**); 1 cm (**P**).

##### Description on MLA.

Vegetative hyphae hyaline to pale brown. Setae absent. Conidiophores 95–150(–195) μm long, 3.5–4.5 μm wide, conidiogenous cells 25–35 × 3.5–4(–4.5) μm, tapering to 1.5 µm just below the collarette, integrated, terminal, polyphialidic, usually cylindrical, pale brown to subhyaline, smooth-walled; collarette funnel-shaped, 3.5–4(–4.5) μm wide, 1.5–2 μm deep. Conidia in slimy droplets, of two types, narrower and longer (13–)13.5–15.5(–17) × 2.5–3 µm (mean ± SD = 14.4 ± 0.9 × 2.7 ± 0.2 µm), usually slightly wider and shorter 11–13 × 2.5–3.5 µm (mean ± SD = 12.0 ± 0.7 × 3.1 ± 0.3 µm), falcate, asymmetrical, hyaline, with simple setulae 3.5–5.5(–7.5) μm long at both ends.

##### Culture characteristics.

On CMD colonies 80–85 mm diam, circular, flat, margin fimbriate, aerial mycelium restricted mainly to the centre and margin of the colony, sparsely lanose, floccose centrally becoming mucoid towards the margin, cobwebby at the margin, colony centre whitish, pale brown to creamy towards the margin, pale brown pigment diffusing from the centre of the colony to the agar; reverse creamy. On MLA colonies 65–70 mm diam, circular, slightly raised, margin filiform, lanose, floccose, colony centre whitish becoming brown-grey towards the margin with a brown outer zone of submerged growth, pale brown pigment diffusing to the agar; reverse dark brown. On OA colonies 89–95 mm diam, circular, raised, margin filiform, aerial mycelium occasionally reduced or absent, colonies similar to those on MLA, lanose, floccose, locally mucoid and smooth or cobwebby, whitish becoming dark grey at the margin, a dark brown to burgundy brown pigment diffusing to the agar; reverse dark grey. On PCA colonies 78–89 mm diam, circular, flat to slightly raised, margin entire to weakly filiform, lanose, floccose, occasionally locally mucoid and smooth or with sparse decumbent aerial hyphae, cobwebby at the margin, whitish becoming brown towards the margin; reverse olivaceous brown. Sporulation on MLA, OA, CMD after 8 wk.

##### Other specimen examined.

France – Ariège • Pyrénées Mts., Rimont, Le Baup stream, ca. 1.5 km from the village along D18 road; alt. 550 m; 12 Jun. 2009; on submerged wood of *Fraxinus excelsior*; J. Fournier leg.; J.F. 09153 (PRA-16320, culture CBS 127692) • *Ibid*.; 23 May 2008; on submerged wood of *Alnus glutinosa*; J. Fournier & M. Delpont leg.; J.F. 08124 (PRA-16321, culture CBS 126573). United Kingdom • Liverpool, University Campus Liverpool; 1992; on submerged dead leaf in a pool; G.L. Hennebert leg.; (culture MUCL 34876).

##### Habitat and distribution.

All four isolates analysed in this study originated from the freshwater environment and occurred on decaying wood or leaves of *Alnus glutinosa*, *Fraxinus excelsior* and other unidentified hosts. Based on the BLASTn search of the ITS sequence of *C. paniculata* in GenBank, two isolates from roots of *Elymus mollis* (ITS: KU838460, KU839605, David et al. 2016), a native beach grass on the USA Pacific Northwest coast, and one environmental soil sample from ancient woodland enclosing a conifer plantation in the United Kingdom (ITS: KM374380, Johnson et al. 2014) showed 100 % sequence similarity. Based on these records, *C. paniculata* is known from the north temperate region in Europe in France and United Kingdom and North America in USA, Oregon.

##### Notes.

Among known *Codinaea* species, *C. assamica* is similar to *C. paniculata*, but differs by slightly longer (14.6–16.8 × 2.6–2.8 µm) conidia with longer (9.6–12.8 µm) setulae (Hughes and Kendrick 1968) and formation of polyphialides in vivo. *Dictyochaeta terminalis* (Lin et al. 2019) matches *C. paniculata* in monophialidic conidiogenous cells formed in vivo and aseptate conidia, which are slightly longer and wider (14.7–20.7 × 2.9–4.2 µm). The ITS sequences of examined strains of *C. paniculata* exhibit 99.94–100 % similarity; their comparison with ITS sequences of the closely related *C. assamica*CBS 242.66 (MH858788) and *D. terminalis* GZCC 18-0085 (MN104613) showed 89.7 % and 89.85 % similarity, respectively.

#### Paragaeumannomyces

Taxon classificationFungiChaetosphaerialesChaetosphaeriaceae

Matsush., Matsush. Mycol. Mem. 10: 156. (2003) [2001]. Emend. Réblová & A. N. Miller.

C591DECF-7F55-5BBF-90E8-6BD0078A8660

##### Type species.

*Paragaeumannomyces sphaerocellularis* Matsush., Mycol. Mem. 10: 156. (2003) [2001].

##### Description.

Teleomorph: Ascomata perithecial, non-stromatic, superficial, subglobose to conical, solitary, in small groups or aggregated, sometimes collapsing laterally upon drying, ranging from white, yellow-white, light fawn-grey, ginger-brown, reddish-brown, russet to dark brown, papillate, glabrous or setose, setae dark brown, acute, opaque, scattered over entire ascoma and/or clustered around the ostiole, centrum sometimes pink to pale red. Ostiole periphysate. Ascomatal wall three-layered; outer layer composed of thin-walled, globose, subglobose to polyhedral cells, sometimes containing pale purple pigment when fresh; middle layer composed of brick-like, dark brown cells with opaque walls; inner layer of flattened, thin-walled, subhyaline cells. Paraphyses persistent, branching, tapering. Asci unitunicate, 8-spored, cylindrical-fusiform, stipitate, apex with a non-amyloid apical annulus. Ascospores asymmetrical, cylindrical-filiform, slightly tapering towards the basal end, multiseptate, hyaline, occasionally light pink, with negative or positive dextrinoid reaction in Melzer’s reagent. Synanamorphs: Craspedodidymum-like. Conidiophores mononematous, semi-macronematous to micronematous, brown, septate, unbranched or reduced to single conidiogenous cells. Conidiogenous cells phialidic, obclavate or broadly lageniform, brown, with an apical opening; collarettes flared or cup-shaped. Conidia globose, subglobose, subangular to triangular, unicellular, hyaline, with setulae. Chloridium-like. Conidiophores mononematous, macronematous, brown, septate, unbranched. Conidiogenous cells phialidic, cylindrical, subhyaline, elongating percurrently, with an apical opening; collarette indistinct or flared. Conidia globose, ovoid to clavate, unicellular, hyaline, non-setulate, accumulating in slimy droplets. [Characteristics of the synanamorphs adopted from Huhndorf and Fernández (2005)].

##### Notes.

The holotype of *P. sphaerocellularis* (Japan, Schimizu-cho, Wakayama Pref., on decaying twig of unknown broadleaf tree, Apr. 2000, MFC-21077), the type species of *Paragaeumannomyces* (Matsushima 2003), was not available to us. A comparison of its protologue with our specimens and descriptions of other scolecosporous species of *Chaetosphaeria* (Carroll and Munk 1964; Huhndorf and Fernández 2005; Atkinson et al. 2007; Perera et al. 2016), combined with phylogenetic analysis of the ITS-28S sequences of 35 isolates, provided sufficient evidence to consider them congeneric. *Paragaeumannomyces* is proposed as the correct name for this morphologically and phylogenetically well-delimited group of chaetosphaeriaceous fungi. The width of the ascus is sometimes variable even within a single collection depending on the arrangement of ascospores in the sporiferous part, whether they are 2–3-seriate, 4-seriate end-to-end or in a fascicle.

Members of *Paragaeumannomyces* display a wide geographical distribution pattern; they have a predominantly pantropical distribution in Central America and Asia but were also encountered in the subtropical and temperate climate zones of Europe, Japan, New Zealand and North America.

### Key to *Paragaeumannomyces* species

**Table:** 

1	Ascomata almost white, yellowish-white or light fawn-grey when fresh, dark yellow, buff, tawny to dark ginger-brown or yellow-grey when dried, purple pigment absent in cells of the outer ascomatal layer, setae absent	**2**
–	Ascomata reddish, reddish-brown, russet or brown, sometimes with red surface crystals, globose cells in the outer ascomatal layer may contain pale purple pigment when fresh, setae present or absent	**3**
2	Ascomata almost white to yellowish-white, translucent, areolate when fresh, with a distinct black papilla, asci 270–295 × 18.5–20.5 μm, ascospores (3–)5–11-septate, (55–)69–86 × 5–6.5(–7) μm	***P. albidus***
–	Ascomata light fawn-grey, not areolate when fresh, papilla indistinct or absent, asci 215–270 × 11–14 µm, ascospores (7–)11(–13)-septate, 62–88 × 4.5–6 µm	***P. bombycinus***
3	Ascomatal setae present only at the apex or absent	**4**
–	Ascomatal setae present at the apex and also scattered over entire surface of the ascoma, or setae only scattered over ascoma, occasionally absent	**8**
4	Ascomata with red surface crystals, ascospores 7-septate, 80–100 × 3.5–4.2 μm, anamorph craspedodidymum-like, conidia globose to subglobose in a vertical position, with setulae	***P. rubicundus***
–	Ascomata without red surface crystals, ascospores with seven or more septa	**5**
5	Ascomata more than 500 μm diam, setae occasionally absent, ascospores 7-septate, 50–100 × 4.5–6 μm, anamorph craspedodidymum-like, conidia oblate to horizontally oblong with a short abscission scar or frill, without setulae, tropical distribution	***P. lapazianus***
–	Ascomata less than 500 μm diam, setae present at the apex, ascospores with seven or more septa, temperate and subtropical distribution	**6**
6	Ascospores (7–)11–13-septate, (90–)95–123.5 × 4–5(–5.5) μm, asci 210–295 × (16.5–)17–24.5 μm	***P. granulatus***
–	Ascospores with up to 11 septa, 87 μm and shorter	**7**
7	Ascospores 9–11-septate, (58–)60.5–80.5 × (3–)3.5–4.5(–5) μm, asci (134–)140–174(–189) × 11–13(–14) μm	***P. smokiensis***
–	Ascospores (5–)7–9(–11)-septate, (62–)65–87 × (3.5–)4–5.5 μm, asci (185–)195–240 × 12–14.5(–15.5) μm	***P. abietinus***
8	Setae present at the apex and also scattered over entire surface of ascoma, ascospores 7-septate	**9**
–	Setae scattered over entire surface of ascoma, occasionally absent, ascospores with seven or more septa	**10**
9	Ascospores (50.4–)52.5–68 × 3.5–4.5 μm, craspedodidymum- and chloridium-like synanamorphs, conidia without setulae	***P. longisporus***
–	Ascospores (64.5–)68.5–86.5(–88.5) × (3–)3.5–4.5 μm, anamorph unknown	***P. sabinianus***
10	Ascospores 7-septate	**11**
–	Ascospores with more than seven septa	**14**
11	Asci up to 152 μm long	**12**
–	Asci 150 μm and longer	**13**
12	Ascomatal setae 60–75 μm long, ascospores 65–75 × 3–4 µm, asci 123–140 × 10–11 µm, craspedodidymum-like anamorph, conidia without setulae, purple-pigmented aleuriospore-like cells present in culture	***P. panamensis***
–	Ascomatal setae 38–47 μm long, ascospores 63.3–75 × 2.3–3.7 µm, asci 120–152 × 10.7–13.3 µm, anamorph unknown	***P. garethjonesii***
13	Ascospores (50–)60–100(–150) × 3–3.75(–4.5) µm, asci (150–)180–250(–350) × 10–20(–27) µm, craspedodidymum-like anamorph, conidia with or without setulae, some strains also with a chloridium-like synanamorph	***P. raciborskiis. lat.*** (*fide*Huhndorf and Fernández 2005; as *Paragaeumannomyces* sp. 1–4 in the phylogeny, Fig. 2)
–	Ascospores (57.5–)60–73(–75) × (3.5–)4–4.5(–5) μm, asci (152–)174–221(–227) × 10.5–15(–20) μm, anamorph unknown	***P. elegans***
14	Ascospores 5–10-septate, 65–90 × 3–4 µm, asci 105–125 × 10–12.5 µm	***P. sphaerocellularis***
–	Ascospores 13–16-septate, 50–65 × 2–4 μm, asci 70–100 × 10–13 μm	***P. raciborskiis. str.*** (*fide*Penzig and Saccardo 1897; Carroll and Munk 1964)

#### Paragaeumannomyces abietinus

Taxon classificationFungiChaetosphaerialesChaetosphaeriaceae

Réblová, J. Fourn. & A.N. Mill. sp. nov.

82B6D25F-DF0B-5E6E-8C85-4882CF07D200

836527

Figure 4

##### Typification.

France – Ariège • Pyrénées Mts., Ustou, Cirque de Cagateille, path up to the La Hillette lake, mixed *Abies* forest; alt. 1550 m; 18 Jul. 2018; on decaying wood of a trunk of *Abies alba*; J. Fournier leg.; J.F. 18057 (**holotype**: PRA-16323!, ex-type culture CBS 145351).

##### Etymology.

Referring to the host *Abies alba*.

##### Description on the natural substrate.

Teleomorph: Ascomata perithecial, non-stromatic, superficial, solitary or in small groups, 350–450 μm diam, 360–500 μm high, broadly conical, collapsing laterally upon drying, finely roughened, dark reddish-brown, glabrous except for the black conical papilla, with dark brown, stiff, acute setae, 32–40 × 3–4 μm, densely clustered around the ostiole; centrum pink. Ostiole periphysate. Ascomatal wall leathery, three-layered. Outer layer of textura angularis, 33–58 μm thick, consisting of thin-walled, globose, subglobose to polyhedral, dark ginger-brown to reddish-brown cells, 6.5–11 μm diam, grading into smaller cells towards the exterior. Middle layer of textura prismatica, 18–25 μm thick, composed of thick-walled, polyhedral, dark brown, melanised cells. Inner layer of textura prismatica, 10–15 μm thick, composed of thin-walled, flattened and elongated hyaline cells. Paraphyses abundant, hyaline, sparsely branched, septate, 4.5–7 μm wide, tapering to 2–2.5 μm, longer than the asci. Asci (185–)195–240 × 12–14.5(–15.5) μm (mean ± SD = 209.2 ± 12.0 × 14.1 ± 0.8 μm), (145–)155–205 µm (mean ± SD = 172.6 ± 14.3 μm) long in the sporiferous part, cylindrical-fusiform, stipitate, apically rounded, ascal apex non-amyloid with a distinct apical annulus 3–3.5 μm wide, 2–3 μm high. Ascospores (62–)65–87 × (3.5–)4–5.5 μm, filiform to cylindrical, straight or slightly curved to sigmoid, hyaline, light pink in mass, with dextrinoid reaction in Melzer’s reagent turning reddish-brown except for the end cells which remain hyaline, (5–)7–9(–11)-septate, septa often unevenly distributed, not constricted or slightly constricted at the septa, especially at the septa above and below the middle, asymmetrical, rounded at the apical end, tapering towards the basal end, with one or two guttules in each cell, 2–3-seriate or 4-seriate and partially overlapping or 4-seriate forming two fascicles end to end. Anamorph: Unknown.

**Figure 4.**
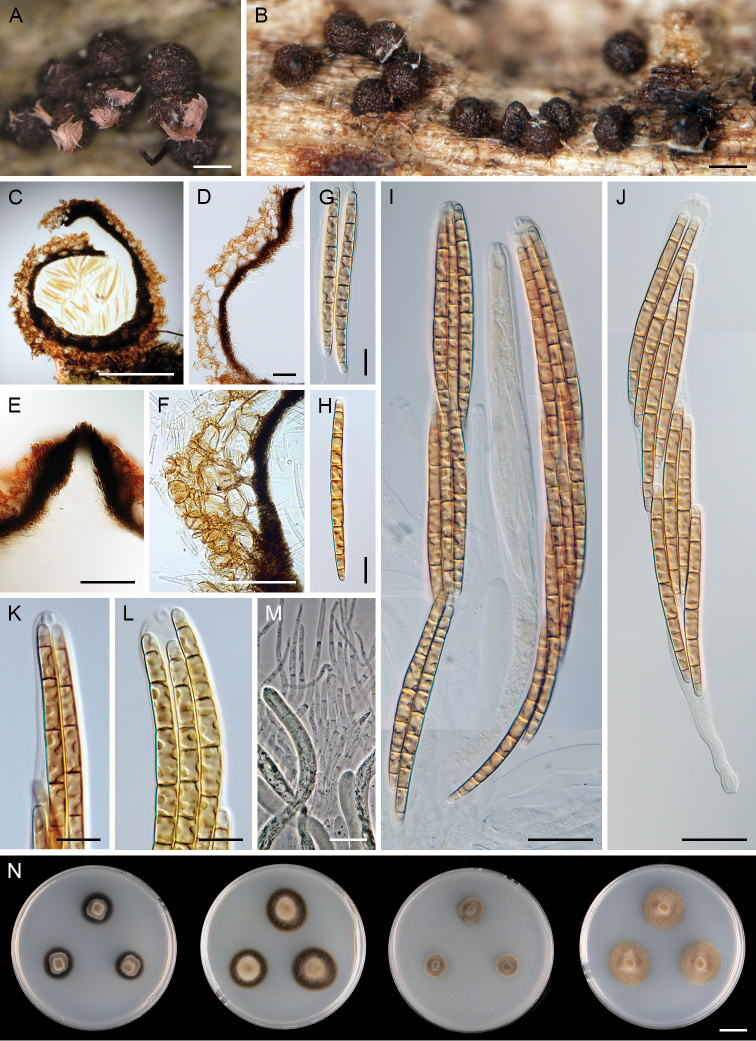
*Paragaeumannomyces abietinus*. **A, B** ascomata **C, D, F** vertical section of ascomal wall **E** vertical section of the ascomal wall and papilla with apical setae **G, H** ascospores **I, J** asci **K, L** ascal apex with apical ring **M** paraphyses **N** colonies on CMD, MLA, OA and PCA after 4 wk (from left to right). Images: PRA-16327 (**A**); CBS 145351 (**B, F–L, N**); PRA-16324 (**C**); PRA-16325 (**D, E, M**). Scale bars: 250 μm (**A, B**); 200 μm (**C**); 50 μm (**D–F**); 20 μm (**I, J, M**); 10 μm (**G, H, K, L**); 1 cm (**N**).

##### Culture characteristics.

On CMD colonies 10–11 mm diam, circular, slightly convex, margin entire to weakly fimbriate, lanose, beige-brown with a dark brown outer zone of submerged growth, dark brown pigment diffusing from the colony margin to agar; reverse dark brown to black. On MLA colonies 12–15 mm diam, circular, slightly convex, margin entire, lanose, floccose, cobwebby at the margin, beige-brown with a dark brown outer zone of submerged growth, brown pigment diffusing from the colony margin to agar; reverse dark brown. On OA colonies 8–9 mm diam, circular, convex, margin entire, lanose, beige-brown, with a paler outer ring; reverse brown. On PCA colonies 14–15 mm diam, circular, convex, margin entire, lanose, floccose, cobwebby towards the margin, beige, pale brown towards the margin; reverse brown. Sporulation absent on all media, even after prolonged incubation (> 3 mo).

##### Other specimen examined.

Ukraine • Carpathian Mts., Kvasi, Bliznica near Rachiv, right bank of the upper flow of the Tisa river; alt. 1000 m; 28 Jun. 1997; on decaying wood of *Abies alba*; M. Réblová leg.; M.R. 946 (PRA-16324). • *Ibid.*; M.R. 947 (PRA-16325). • *Ibid.*; M.R. 959 (PRA-16326). Ukraine • Carpathian Mts., Massif Boržava, Guklivij; 21 Jul. 1998; on decaying wood of *Abies alba*; M. Réblová leg.; M.R. 1309 (PRA-16327).

##### Habitat and distribution.

All specimens of *P. abietinus* occur on decaying wood of *Abies alba*. The species has been collected in mountain areas and is known in Europe in France and Ukraine.

##### Notes.

Attempts to cultivate this species were unsuccessful for the Ukrainian specimens; the ascospores germinated over five days with long inflated germ tubes from both ends but did not grow after isolation on agar medium. The axenic culture derived from the ascospore isolate of the French material yielded sterile mycelium only.

*Paragaeumannomyces abietinus* is similar to *P. rubicundus* and *P. lapazianus* in reddish-brown ascomata, the arrangement of setae around the ostiole and distribution in the north temperate region. *Paragaeumannomyces rubicundus* (Huhndorf and Fernández 2005) can be distinguished from the present species in having 7-septate, longer (80–100 × 3.5–4.2 μm) ascospores and red surface crystals; *P. lapazianus* has 7-septate ascospores and a broader range of ascospore lengths including shorter and broader ascospores [(45–)50–100(–120) × (3–)4.5–6(–7) µm] and larger ascomata [(400–)500–950 µm diam, 525–825(–1025) µm high]. In the ITS-28S phylogenetic tree (Fig. 2), *P. abietinus* was clustered with *P. granulatus* (New Zealand) and *P. smokiensis* (USA). These species are morphologically highly similar; they share glabrous, dark brown to reddish-brown ascomata except for the black papilla containing short, appressed setae, and ascospores exhibiting a dextrinoid reaction in Melzer’s reagent. *Paragaeumannomyces granulatus* differs from *P. abietinus* in longer [(90–)95–123.5 μm], (7–)11–13-septate ascospores, while *P. smokiensis* is distinguished from the latter species by shorter and slightly narrower asci [(134–)140–174(–189) × 11–13(–14) μm] and ascospores with more septa (9–11-septate).

#### Paragaeumannomyces albidus

Taxon classificationFungiChaetosphaerialesChaetosphaeriaceae

(T.J. Atk., A.N. Mill. & Huhndorf) Réblová & A.N. Mill. comb. nov.

6C146A6D-0A83-5132-B176-861389DA2691

836528

Figure 5

##### Basionym.

*Chaetosphaeria albida* T.J. Atk., A.N. Mill. & Huhndorf, New Zealand J. Bot. 45: 688. 2007.

##### Specimens examined.

New Zealand – Tasman • Tasman District, Abel Tasman National Park, Pigeon Saddle point, unpaved road between Tata Beach and Totaranui ca. 10 km NW of Totaranui; 24 Feb. 2003; on decaying wood of *Nothofagus* sp. buried in soil; M. Réblová leg.; M.R. 2605/NZ 76 (PDD 118737). – West Coast • Westland District, Arthur’s Pass National Park, Kelly Shelter ca. 5 km W of Otira, Cockayane Nature Walk, a podocarp-broadleaf forest; 16 Mar. 2003; on decaying wood of a trunk; M. Réblová leg.; M.R. 2840/NZ 351 (PDD 118738). • *Ibid.*; 16 Mar. 2003; on decaying wood and bark of a branch; M. Réblová leg.; M.R. 2843/NZ 355 (PDD 118739).

##### Habitat and distribution.

*Paragaeumannomyces albidus* has been collected on *Metrosideros robusta*, *Metrosideros* sp., *Nothofagus* sp. and other unidentified hosts and is known from New Zealand (Atkinson et al. 2007; this study).

##### Notes.

For additional illustrations and description, see Atkinson et al. (2007).

**Figure 5.**
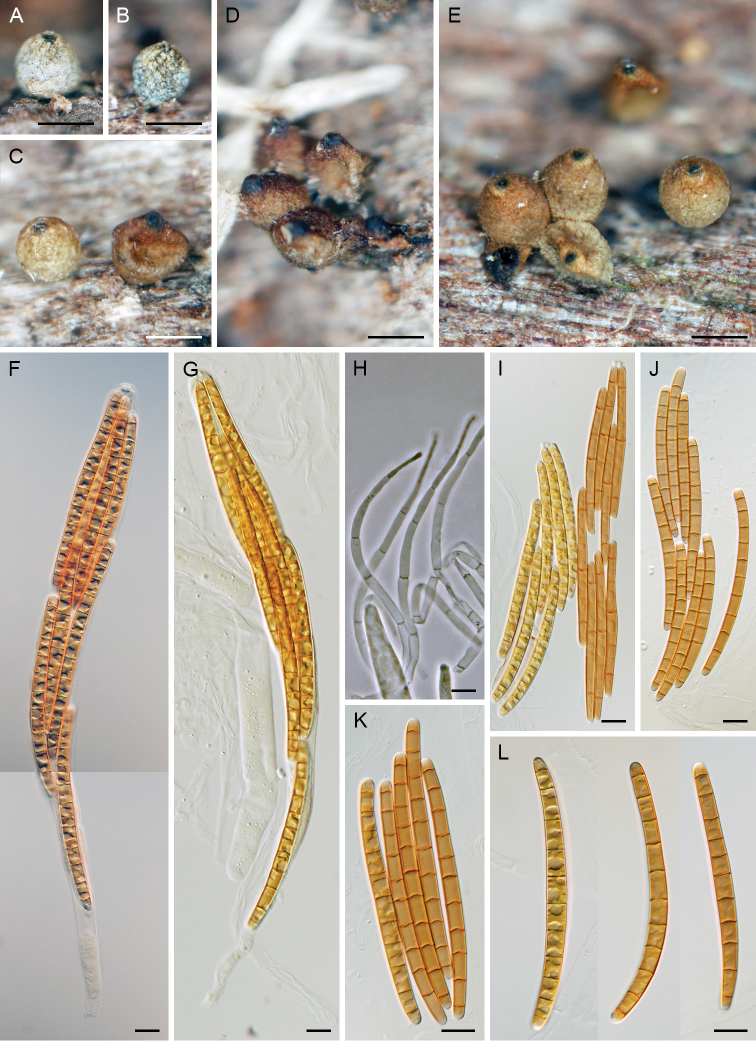
*Paragaeumannomyces albidus* (PDD 118738). **A, B** young ascomata **C–E** mature ascomata **F, G** asci **H** paraphyses **I, J** sporiferous parts of the asci **K, L** ascospores. Scale bars: 250 μm (**A–E**); 10 μm (**F–L**).

*Paragaeumannomyces albidus* is the only species of the genus characterised by a wide range of ascoma colours that change when ascomata are young and fresh or mature and dried. Different colours were used by Atkinson et al. (2007) to distinguish *P. albidus* from closely related *P. bombycinus*. *Paragaeumannomyces albidus* differs from the latter species in having distinctly papillate ascomata, which are almost white, yellowish-white, areolate and translucent when young except for the black papilla (Fig. 5A, B). In older specimens and after drying, ascomata often become laterally pinched, dark yellow, buff, tawny to dark ginger-brown (Fig. 5C–E). The ascomatal wall of *P. albidus* is thicker than that of *P. bombycinus*, with an outer layer containing an external melanised section. In our material, asci were longer than those in the original description, 270–295 × 18.5–20.5 μm long and 155–225 μm long in the sporiferous part vs 220–260 × 16–20 μm *fide*Atkinson et al. (2007). The size and septation of ascospores matched those given in the protologue, though being slightly longer in the upper range: (3–)5–11-septate, (55–)69–86 × 5–6.5(–7) μm vs (5–)7(–12)-septate, (47–)60–80 × 5–7 μm *fide*Atkinson et al. (2007). The ascospores exhibited a dextrinoid reaction in Melzer’s reagent turning reddish-brown except for the tips of the end cells that remain hyaline.

Attempts to isolate our specimens of *P. albidus* in living culture were not successful. Therefore, the DNA was extracted from herbarium material of all three collections, but only ITS1 of PDD 118738 could be amplified and sequenced. Comparison of the ITS1 sequences of our specimen and the holotype of *P. albidus* revealed 100 % similarity (Fig. 2).

#### Paragaeumannomyces bombycinus

Taxon classificationFungiChaetosphaerialesChaetosphaeriaceae

(T.J. Atk., A.N. Mill. & Huhndorf) Réblová & A.N. Mill. comb. nov.

7FEEA223-D70E-50BC-B481-31BA7DE9C121

836529

##### Basionym.

*Chaetosphaeria bombycina* T.J. Atk., A.N. Mill. & Huhndorf, New Zealand J. Bot. 45: 691. 2007.

##### Habitat and distribution.

The species has been collected on decaying wood of *Nothofagus* sp. and is known from New Zealand (Atkinson et al. 2007).

##### Notes.

For description, illustration and holotype information, see Atkinson et al. (2007). Although *P. albidus* and *P. bombycinus* share identical ITS sequences and the size of their ascomata, asci and ascospores considerably overlap, the latter species was distinguished by characters in the ascomatal wall, ascoma appearance and ascospore septation. The ascomata of *P. bombycinus* are light fawn-grey and non-areolate when fresh, the ascomatal wall lacks the external melanised layer or the melanisation is only weakly present, and the black papilla is lacking or indistinct being covered by the outer layer. The ascospores of *P. bombycinus* are (7–)11(–13)-septate compared to (5–)7(–12)-septate ascospores of *P. albidus*. Atkinson et al. (2007) considered the ITS sequence identity uninformative in the light of distinct morphologies between the two species. Another explanation to this peculiar case could be that *P. bombycinus* is a described anomaly within *P. albidus* based on a single collection. More specimens of the “*bombycinus*” phenotype need to be examined, and their ITS and possibly other loci analysed.

#### Paragaeumannomyces elegans

Taxon classificationFungiChaetosphaerialesChaetosphaeriaceae

Réblová & A.N. Mill. sp. nov.

4AAE0649-8AE3-55AD-BEB3-ADA68B12DFBF

836530

Figure 6

##### Typification.

New Zealand – West Coast • Westland District, Mount Aspiring National Park, Haast, Roaring Billy track; 22 Mar. 2005; on decaying wood; M. Réblová leg.; M.R. 3295/NZ 566A (**holotype**: PDD 118740!).

##### Etymology.

*Elegans* (L) elegant, referring to elegant and lovely ascomata adorned with setae.

##### Description on the natural substrate.

Teleomorph: Ascomata perithecial, non-stromatic, superficial, in small groups, often gregarious, 290–350 μm diam, 280–350 μm high, subglobose to slightly conical, dully glossy, brown with a light grey tinge except for the tiny black papilla composed of thick-walled, dark brown cells, ascomata densely setose, setae 28–60 × 4.5–6 μm, stiff, acute, dark brown, thick-walled, opaque. Ostiole periphysate. Ascomatal wall leathery, three-layered. Outer layer of textura angularis, 33–41 μm thick, consisting of thin-walled, globose to subglobose to polyhedral, reddish-brown cells ca. 5–12 μm diam. Middle layer of textura prismatica, 9.5–18 μm thick, composed of thick-walled, polyhedral, elongated, dark brown, melanised cells. Inner layer of textura prismatica, 5–8 μm thick, composed of thin-walled, flattened and elongated hyaline cells. Paraphyses abundant, hyaline, sparsely branched, septate, 3.5–5 μm wide, tapering to ca. 2 μm, longer than the asci. Asci (152–)174–221(–227) × 10.5–15(–20) μm (mean ± SD = 204.8 ± 13.7 × 12.3 ± 1.5 μm), (129–)141–197(–204) μm (mean ± SD = 168.2 ± 17.2 μm) long in the sporiferous part, cylindrical-fusiform, stipitate, apically rounded, ascal apex non-amyloid with a distinct apical annulus 2.5–3 μm wide, 2–2.5 μm high. Ascospores (57.5–)60–73(–75) × (3.5–)4–4.5(–5) μm (mean ± SD = 65.5 ± 3.2 × 4.1 ± 0.2 μm), filiform to cylindrical, straight or slightly curved to sigmoid, hyaline, with negative or very weak dextrinoid reaction in Melzer’s reagent, 7–septate, septa usually obscured by large guttules, not constricted at the septa, asymmetrical, rounded at the apical end, slightly tapering towards the basal end, with one or two guttules in each cell, 2–3-seriate or 3–4-seriate, partially overlapping. Anamorph: Unknown.

**Figure 6.**
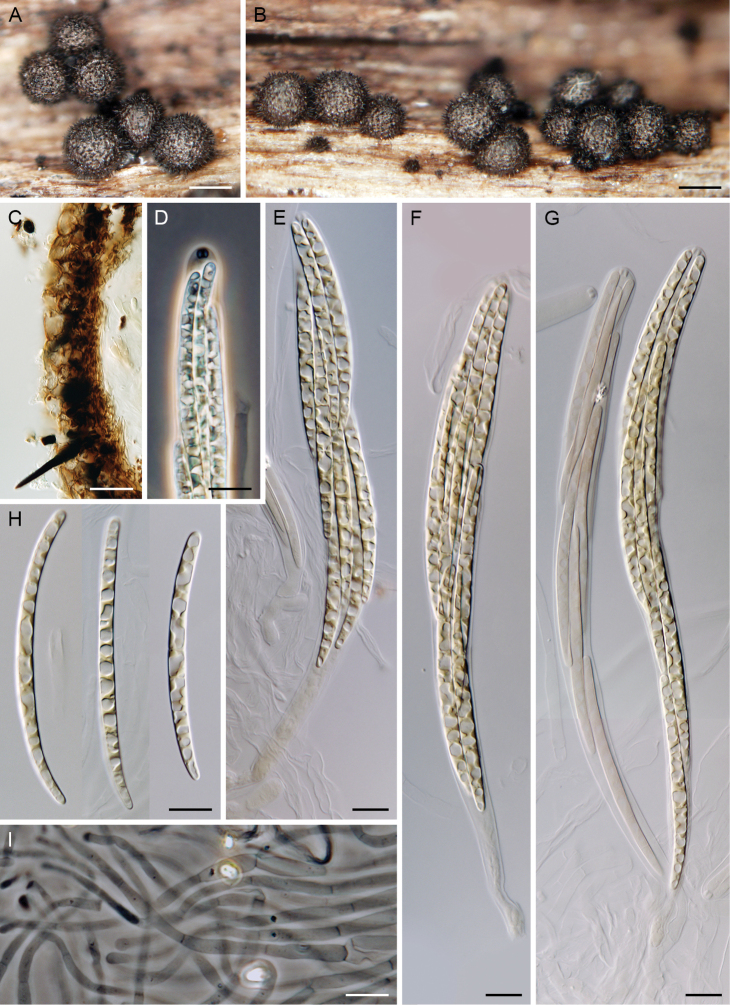
*Paragaeumannomyces elegans*. **A, B** ascomata **C** vertical section of ascomal wall **D** ascal apex with apical annulus **E–G** asci **H** ascospores **I** paraphyses. Images: PDD 118740 (**A, B, D, H, I**); PDD 118741 (**C, E–G**). Scale bars: 250 μm (**A, B**); 20 μm (**C**); 10 μm (**D–I**).

##### Other specimen examined.

New Zealand – Otago • Clutha District, The Catlins, Catlins Coastal Rain Forest Park, MacLennan Range, Lake Wilkie Walk; 17 Mar. 2005; on decaying wood of a branch; M. Réblová leg.; M.R. 3289/NZ 549 (PDD 118742). – West Coast • Westland District, Ship Creek Point, Kahikatea Swamp Forest walk; 8 Mar. 2003; on decaying wood; M. Réblová leg.; M.R. 2819/NZ 329 (PDD 118741). – West Coast • Westland District, Ross, Totara Valley Road, 12 Apr. 2005; on decaying wood; M. Réblová leg.; M.R. 3486/NZ 775 (PDD 118743).

##### Habitat and distribution.

The present species is a saprobe on decaying wood of *Nothofagus* sp. and other unidentified hosts, known from New Zealand (Atkinson et al. 2007; this study).

##### Notes.

*Paragaeumannomyces elegans* is distinguishable from other members of the genus by densely setose, dull glistening brown ascomata with a light grey tinge, which gives them an almost grey appearance when dried. The new species resembles *P. garethjonesii* (Perera et al. 2016) and *P. panamensis* (Huhndorf and Fernández 2005) in 7-septate ascospores and setose ascomata with acute, stiff, opaque setae scattered over the entire surface, but differs from them in larger ascomata, asci and wider ascospores (for a detailed comparison see the key).

Comparison of the ITS sequence of the holotype of *P. elegans* with available *Paragaeumannomyces* sequences revealed 100 % sequence similarity with a specimen PDD 92561 (New Zealand, Taupo, Ohakune, ITS: EUO37895) tentatively identified as *P. raciborskii* (Atkinson et al. 2007) (Fig. 2).

#### Paragaeumannomyces garethjonesii

Taxon classificationFungiChaetosphaerialesChaetosphaeriaceae

(R.H. Perera, Maharachch. & K.D. Hyde) Réblová & A.N. Mill. comb. nov.

2A69DA51-254D-5E22-89E4-1177408D8757

836531

##### Basionym.

*Chaetosphaeria garethjonesii* R.H. Perera, Maharachch. & K.D. Hyde, Mycosphere 7: 1308. 2016.

##### Habitat and distribution.

*Paragaeumannomyces garethjonesii* was collected on a Fabaceae seed pod and is known from Asia in Thailand (Perera et al. 2016).

##### Notes.

For description, illustration and holotype information see Perera et al. (2016). *Paragaeumannomyces garethjonesii* resembles *P. panamensis* (Huhndorf and Fernández 2005) in size of ascomata, which are the smallest (up to 250 μm diam and 270 μm high) within the genus, setae scattered over the entire ascoma, overlapping length of their asci and 7-septate ascospores, but it differs by shorter (38–47 μm) setae, slightly wider (10.7–13.3 µm) asci and the absence of aleuriospore-like cells in culture (for a detailed comparison see the key).

#### Paragaeumannomyces granulatus

Taxon classificationFungiChaetosphaerialesChaetosphaeriaceae

Réblová & A.N. Mill. sp. nov.

F71FA226-0E8C-56F1-889D-E6C6B4D2D148

836532

Figure 7

##### Typification.

New Zealand – West Coast • Westland District, Hokitika, Mananui Point, Lake Mahinapua, Swimmers Beach walks; 5 Mar. 2003; on decaying wood; M. Réblová leg.; M.R. 2715/NZ 216 (**holotype**: PDD 118744!, ex-type culture ICMP 15133).

##### Etymology.

*Granulum* (L), granule, small grain, diminutive of *granum*, referring to the roughened surface of the ascomatal wall composed of globose cells, which appears granulose in the surface view.

##### Description on the natural substrate.

Teleomorph: Ascomata perithecial, non-stromatic, superficial, solitary or in small groups, 380–495 μm diam, 415–530 μm high, subglobose to conical, finely roughened, dark brown to dark reddish-brown, sometimes with irregular reddish colour except for the black papilla; papilla composed of dark brown, thick-walled, cylindrical to subulate, apically narrowly rounded soft setae; centrum pink. Ostiole periphysate. Ascomatal wall leathery, three-layered. Outer layer of textura angularis, 95–115 μm thick, consisting of thin-walled, globose to subglobose ginger-brown cells ca. 27–33 μm diam, grading into smaller cells 8–16 μm diam. Middle layer of textura prismatica, 14–21 μm thick, composed of thick-walled, polyhedral, elongated, dark brown, melanised cells. Inner layer of textura prismatica, 7–12 μm thick, composed of thin-walled, flattened and elongated hyaline cells. Paraphyses abundant, hyaline, sparsely branched, septate, 3.5–5 μm wide, tapering to 2–2.5 μm, longer than the asci. Asci 210–295 × (16.5–)17–24.5 μm (mean ± SD = 239.7 ± 15.5 × 20.3 ± 2.1 μm), 165–200(–250) μm (mean ± SD = 184.7 ± 10.3 μm) long in the sporiferous part, cylindrical-fusiform, stipitate, apically rounded, ascal apex non-amyloid with a distinct apical annulus 3.5–4 μm wide, 2.5–3(–3.5) μm high. Ascospores (90–)95–123.5 × 4–5(–5.5) μm (mean ± SD = 101.4 ± 10.2 × 4.8 ± 0.4 μm), filiform to cylindrical, straight or slightly curved to sigmoid, hyaline to very light pink, light pink-brown in mass, with dextrinoid reaction in Melzer’s reagent turning reddish-brown except for the end cells which remain hyaline, (7–)11–13-septate, septa often unevenly distributed, not constricted or slightly constricted at the septa, asymmetrical, broadly rounded at the apical end, tapering and narrowly rounded at the basal end, with one or two guttules in each cell, 2–3-seriate or 4-seriate and partially overlapping. Anamorph: Unknown.

**Figure 7.**
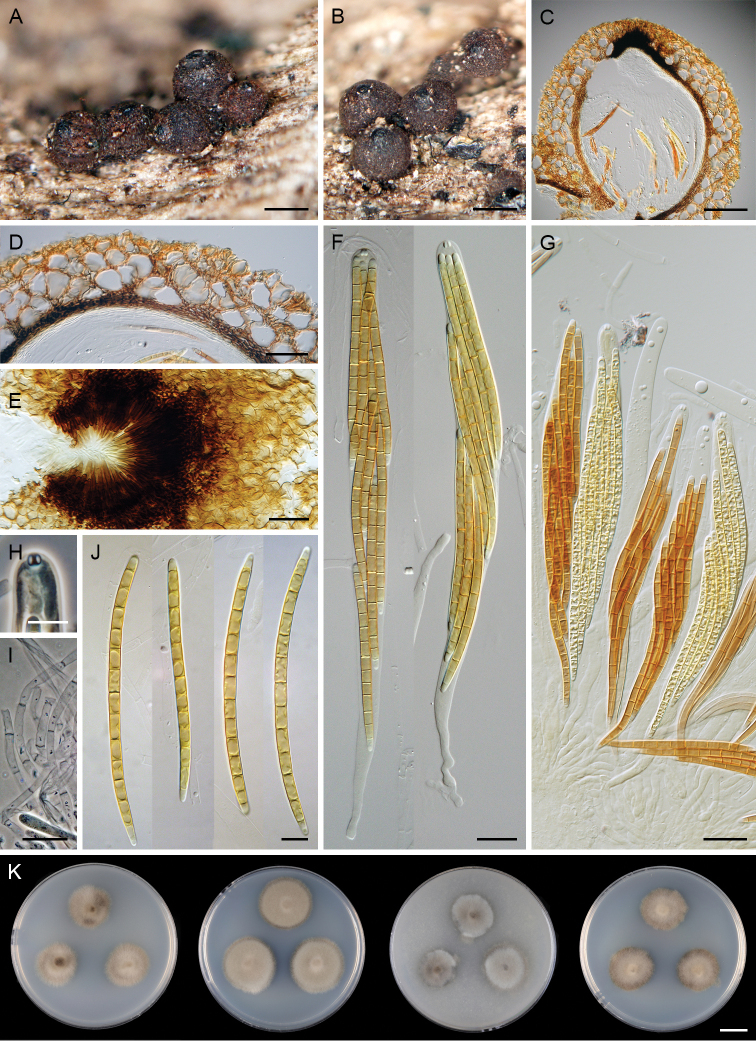
*Paragaeumannomyces granulatus* (PDD 118744). **A, B** ascomata **C, D** vertical section of ascomal wall **E** papilla and the upper part of ascomal wall in surface view **F, G** asci **H** ascal apex with apical annulus **I** paraphyses **J** ascospores **K** colonies on CMD, MLA, OA and PCA after 4 wk (from left to right). Scale bars: 250 μm (**A, B**); 100 μm (**C**); 25 μm (**G**); 20 μm (**D–F, I**); 10 μm (**H, J**); 1 cm (**K**).

##### Culture characteristics.

On CMD colonies 14–16 mm diam, circular, convex, margin fimbriate, lanose, grey-brown, reverse dark brown to almost black. On MLA colonies 19–20 mm diam, circular, raised, margin entire to weakly fimbriate, lanose, beige-brown, with a dark brown outer zone, reverse dark brown to almost black. On OA colonies 13–16mm diam, circular, raised, margin weakly fimbriate, lanose, beige-grey becoming grey towards the periphery, reverse dark brown to almost black. On PCA colonies 15–17 mm diam, circular, slightly convex, margin weakly fimbriate, lanose, beige, pale brown at the margin, reverse black. Sporulation absent on all media.

##### Other specimen examined.

New Zealand – Auckland • Auckland district, Waitakere Ranges Nature Reserve, Anawhata Road; 24 Apr. 2005; on decaying wood; M. Réblová leg.; M.R. 3543/NZ 838 (PDD 118745).

##### Habitat and distribution.

A saprobe on decaying wood, known from New Zealand.

##### Notes.

*Paragaeumannomyces granulatus* most closely resembles *P. abietinus* in the ascoma appearance, pink content of the ascoma centrum, ascospores with usually more than seven septa and positive dextrinoid reaction in Melzer’s reagent but both species are separated by size of asci and ascospores. The ascospores of *P. abietinus* are (5–)7–9(–11)-septate and shorter [(62–)65–87 μm] and asci are shorter and narrower [(185–)195–240 × 12–14.5(–15.5) μm].

#### Paragaeumannomyces lapazianus

Taxon classificationFungiChaetosphaerialesChaetosphaeriaceae

(G.C. Carroll & Munk) Réblová & A.N. Mill. comb. nov.

647B4995-143D-5A58-8688-B841BCDEBCC5

836533

≡ Chaetosphaeria lapaziana (G.C. Carroll & Munk) F.A. Fernández & Huhndorf, Fung. Diver. 18: 49. 2005.

##### Basionym.

*Lasiosphaeria lapaziana* G.C. Carroll & Munk, Mycologia 56: 90. 1964.

##### Habitat and distribution.

*Paragaeumannomyces lapazianus* is common on decaying wood in the neotropics and is known from the Caribbean in Puerto Rico and Jamaica, from Central America in Costa Rica, and from South America in French Guiana (Carroll and Munk 1964; Huhndorf and Fernández 2005).

##### Notes.

For description, illustration and holotype information see Carroll and Munk (1964) and Huhndorf and Fernández (2005). *Paragaeumannomyces lapazianus* has 7-septate ascospores and the largest ascomata in the genus, (400–)500–950 μm diam and 525–825(–1025) μm high *fide*Huhndorf and Fernández (2005), and forms a craspedodidymum-like anamorph in vitro. The anamorph is characterised by inflated, pigmented conidiogenous cells with a flared collarette and oblate to horizontally oblong conidia with a short abscission scar or frill and without setulae.

#### Paragaeumannomyces longisporus

Taxon classificationFungiChaetosphaerialesChaetosphaeriaceae

(Sacc.) Réblová & A.N. Mill. comb. nov.

6E1521E1-E431-5840-BA60-37AE8FC6259A

836534

Figure 8

≡ Sphaeria longispora Ellis, Bull. Torrey Bot. Club 6: 135. 1877 non Currey 1859 nec Karsten 1873. (Nom. illegit., Art. 53.1)
≡ Ceratostomella longispora (Sacc.) Cooke, Grevillea 17: 50. 1889.
≡ Chaetosphaeria longispora (Sacc.) P.M. Kirk, Index Fung. 120: 1. 2014.
= Lasiosphaeria ellisii M.E. Barr, Mycotaxon 46: 48. 1993.
≡ Chaetosphaeria ellisii (M.E. Barr) Huhndorf & F.A. Fernández, Fung. Diver. 19: 27. 2005.

##### Basionym.

*Ophioceras longisporum* Sacc., Syll. fung. 2: 360. 1883.

##### Specimens examined.

USA – Tennessee • Cocke Co., Great Smoky Mountains National Park, Cosby, Cosby Nature Trail; alt. 716 m; 23 Mar. 2007; on decaying wood; A.N. Miller, P. Chaudhary & H.A. Raja leg.; A.N.M. 1134 (ILLS00121385). • *Ibid.*; 19 Jul. 2007; T.J. Atkinson & P. Chaudhary leg.; A.N.M. 1250 (ILLS00121386).

##### Habitat and distribution.

The species occurs on decaying wood and is known from the north temperate region in the USA (Indiana, New Jersey, North Carolina, South Carolina, Tennessee) (Barr 1993; Huhndorf and Fernández 2005; this study).

##### Notes.

**Figure 8.**
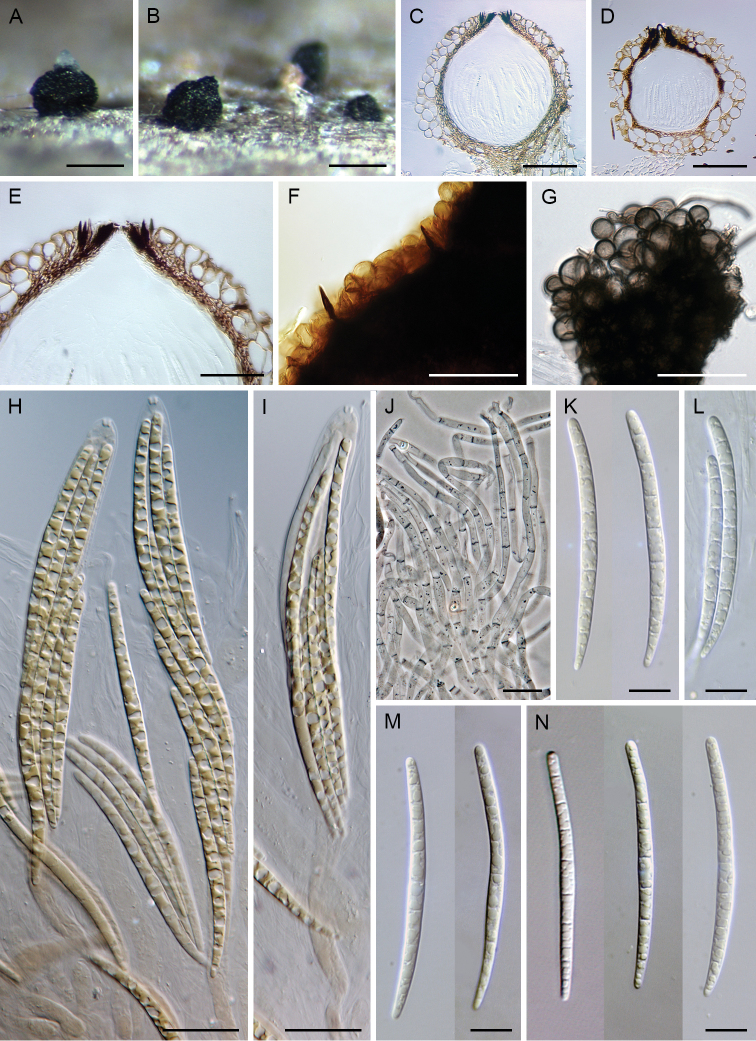
*Paragaeumannomyces longisporus*. **A, B** ascomata **C, D** vertical section of ascomal wall **E** vertical section of ascomal wall and papilla with apical of setae **F** ascomal wall with setae **G** globose cells of the outer layer of the ascomal wall **H, I** asci **J** paraphyses **K–N** ascospores. Images: ILLS00121385 (**A, B, G**); S.M.H. 3860 (**C, E, M**); S.M.H. 2519 (**D**); ILLS00121386 (**F, H–J, K**); S.M.H. 2758 (**L**); S.M.H. 3809 (**N**). Scale bars: 250 μm (**A–D**); 50 μm (**E–G**); 20 μm (**H–J**); 10 μm (**K–N**).

For description and illustration, refer to Barr (1993), and Huhndorf and Fernández (2005). Our specimens of *P. longisporus* match well the fungus described and illustrated by Barr (1993) based on the holotype of *Sphaeria longispora* (Ellis 1877), only the asci are longer and agree with the measurements given by Huhndorf and Fernández (2005). Based on examination of our material (Fig. 8), ascomata are reddish-brown, subglobose to globose, with three-layered wall, setose, setae dark brown, acute, scattered over entire ascomatal surface and also aggregated around the ostiole, asci (140.5–)165–183 × 10.5–12.5 μm and (114–)133–157.5 μm long in the sporiferous part, ascospores (50.5–)52.5–68 × 3.5–4.5 μm, 7-septate, asymmetrical, tapering towards the basal end, with a negative or very weak dextrinoid reaction in Melzer’s reagent.

*Sphaeria longispora* (Ellis 1877) is a later homonym of *S. longispora* (Currey 1859) and *S. longispora* (Karsten 1873). Barr (1993) revised the holotype of *S. longispora* Ellis (USA, New Jersey, Newfield, on fallen branch of *Kalmia latifolia*, 20 Jul 1874, J.B. Ellis, NY) and concluded that the fungus is better placed in *Lasiosphaeria* due to filiform, septate ascospores and setose ascomata and proposed a replacement name, *Lasiosphaeria ellisii* as a nomen novum. This species was later transferred to *Chaetosphaeria* by Huhndorf and Fernández (2005) as *Ch. ellisii*. Kirk (2014) considered the first combination of *S. longispora* in *Ophioceras* by Saccardo (1883) as the earliest legitimate name of the taxon in the same rank (Art. 41.3) to replace *Sphaeria longispora* Ellis. *Ophioceras longisporum* Sacc. is, therefore, a basionym for all future combinations. Kirk (2014) proposed a new combination *Chaetosphaeria longispora* but erroneously cited *S. longispora* as the basionym, which does not affect the valid publication of the new combination (Art. 41.8c).

#### Paragaeumannomyces panamensis

Taxon classificationFungiChaetosphaerialesChaetosphaeriaceae

(Huhndorf & F.A. Fernández) Réblová & A.N. Mill. comb. nov.

A7433647-E7EC-5742-9980-66B243B62468

836535

##### Basionym.

*Chaetosphaeria panamensis* Huhndorf & F.A. Fernández, Fung. Diver. 19:33. 2005.

##### Habitat and distribution.

*Paragaeumannomyces panamensis* has been collected on decaying wood of *Pinus* sp. and an unidentified host, and it is known from Asia in Thailand and Central America in Panama (Huhndorf and Fernández 2005; Perera et al. 2016).

##### Notes.

For description, illustrations and holotype information see Huhndorf and Fernández (2005) and Perera et al. (2016). *Paragaeumannomyces panamensis* is similar to *P. sphaerocellularis* in ascoma and is more or less comparable in size of asci, but differs by shorter, always 7-septate ascospores and occurrence in the tropics. For detailed comparison, see notes to *P. sphaerocellularis*.

#### Paragaeumannomyces raciborskii

Taxon classificationFungiChaetosphaerialesChaetosphaeriaceae

(Penz. & Sacc.) Réblová & A.N. Mill. comb. nov.

65ADA667-339C-51B9-9101-DFEEDA6816E9

836536

≡ Lasiosphaeria raciborskii (Penz. & Sacc.) G.C. Carroll & Munk, Mycologia 56: 91. 1964.
≡ Chaetosphaeria raciborskii (Penz. & Sacc.) F.A. Fernández & Huhndorf, Mycol. Res. 108: 29. 2004.

##### Basionym.

*Ophiochaeta raciborskii* Penz. & Sacc., Malpighia 11: 406. 1897.

##### Habitat and distribution.

*Paragaeumannomyces raciborskii* has been collected on culms of *Chusquea* bamboo and other unidentified bamboo species, on palm wood and fruit, and decaying wood of unknown trees. The species has a pantropical geographical distribution and is probably the most commonly encountered species of the genus; it is known from Indonesia in Java and Central America in Costa Rica (Penzig and Saccardo 1897; Carroll and Munk 1964). Other collections published under this name, which may represent different species, originate from Asia in Thailand, the Caribbean in Cuba, Jamaica and Puerto Rico, Central America in Costa Rica and Panama, and South America in Ecuador, French Guiana and Venezuela (Huhndorf and Fernández 2005).

##### Notes.

For descriptions and illustrations, see Carroll and Munk (1964). The holotype of *P. raciborskii* (Penzig and Saccardo 1897) originates from decaying wood in Java. In the protologue, the species was described with black, setose ascomata 250 μm diam, short-stipitate asci 130–150 × 9–10 μm and hyaline, multiseptate ascospores 60–70 × 3 μm. Carroll and Munk (1964) redescribed the species based on the holotype and an additional collection from Costa Rica as having setose, reddish-brown ascomata 250–300 μm diam, 13–16-septate ascospores 50–65 × 2–4 μm, and asci 70–100 × 10–13 μm. Huhndorf and Fernández (2005) introduced a broader species concept of *P. raciborskii*, which was based on numerous specimens of a tropical geographical distribution originating mainly from Central and South America. The species was characterised by setose, reddish, russet or brown ascomata (150–)200–450 μm diam with stiff, dark setae scattered over the entire ascoma or absent in some specimens, 7-septate ascospores (50–)60–100(–150) × 3–3.75(–4.5) μm, and short-stipitate asci (150–)180–250(–350) × 10–20(–27) μm.

*Paragaeumannomyces raciborskiifide*Huhndorf and Fernández (2005) shows a high degree of ITS sequence variability accompanied by a low phenotypic plasticity, which resulted in the species being polyphyletic and segregated into four lineages labelled *Paragaeumannomyces* sp. 1–4 (Fig. 2). Two anamorphic craspedodidymum-like morphotypes with and without setulae and a chloridium-like synanamorph have been experimentally linked to several strains of *P.* ‘*raciborskii*’ by Huhndorf and Fernández (2005). Although the in vitro anamorphic characters seem promising in becoming another important set of diagnostic features to distinguish species of *Paragaeumannomyces*, isolated strains often form sterile mycelium in vitro or they are difficult to isolate into living culture.

#### Paragaeumannomyces rubicundus

Taxon classificationFungiChaetosphaerialesChaetosphaeriaceae

(Huhndorf & F.A. Fernández) Réblová & A.N. Mill. comb. nov.

E2E5755E-C058-5925-9B0B-B3EDFA192CA1

836537

##### Basionym.

*Chaetosphaeria rubicunda* Huhndorf & F.A. Fernández, Fung. Diver. 19: 39. 2005.

##### Habitat and distribution.

*Paragaeumannomyces rubicundus* occurs on decaying wood and is known from the Caribbean in Puerto Rico and from Central America in Costa Rica (Huhndorf and Fernández 2005).

##### Notes.

For description, illustration and holotype information see Huhndorf and Fernández (2005). *Paragaeumannomyces rubicundus* is distinguished from other species of the genus by ascomata with red surface crystals not dissolving in water, 3 % KOH or lactophenol. Similar to *Paragaeumannomyces* sp. 4 (S.M.H. 3119), the craspedodidymum-like anamorph forms conidia with three setulae.

#### Paragaeumannomyces sabinianus

Taxon classificationFungiChaetosphaerialesChaetosphaeriaceae

Réblová & A.N. Mill. sp. nov.

62B7969B-14E2-5D03-97B0-72912621B66F

836538

Figure 9

##### Typification.

USA – Tennessee • Sevier Co., Great Smoky Mountains National Park, Twin Creeks, Twin Creeks Nature Trail, near ATBI plot; alt. 549 m; 11 Oct. 2006; on decaying wood; A.N. Miller & P. Chaudhary leg.; A.N.M. 1011 (**holotype**: ILLS00121384!).

##### Etymology.

The species epithet is proposed in honour of Sabine M. Huhndorf for her contribution to mycology and studies in *Chaetosphaeria*.

##### Description on the natural substrate.

Teleomorph: Ascomata perithecial, non-stromatic, superficial, usually solitary or in small groups, 250–300(–400) μm diam, 280–320 μm high, subglobose to broadly conical, rarely collapsing laterally upon drying, finely roughened, dark reddish-brown except for a black indistinct papilla, setose, setae 30–41.5 × 4–5 μm, dark brown, stiff, acute, scattered over entire ascoma, shorter and narrower setae 16.5–35 × 2.5–3 μm densely aggregated around the ostiole. Ostiole periphysate. Ascomatal wall leathery, three-layered. Outer layer of textura angularis, 32–53 μm thick, consisting of thin-walled, globose to subglobose, dark orange-brown to reddish-brown cells, 11.5–28 μm diam. Middle layer of textura prismatica, 12–22 μm thick, composed of thick-walled, polyhedral, dark brown, melanised cells. Inner layer of textura prismatica, 3–5 μm thick, composed of thin-walled, flattened and elongated hyaline to subhyaline cells. Paraphyses abundant, hyaline, sparsely branched, septate, 3.5–4.5(–6) μm wide, tapering to 2–2.5 μm, longer than the asci. Asci (154–)161–189 × (11–)12.5–14.5(–15.5) μm (mean ± SD = 174.2 ± 8.7 × 13.0 ± 0.8 μm), (130–)144–165 µm (mean ± SD = 155.2 ± 8.3 μm) long in the sporiferous part, cylindrical-fusiform, stipitate, apically broadly rounded to obtuse, ascal apex non-amyloid with a distinct apical annulus 2.5–3 μm wide, 1.5–2 μm high. Ascospores (64.5–)68.5–86.5(–88.5) × (3–)3.5–4.5 μm, (mean ± SD = 79.1 ± 5.3 × 4.0 ± 0.3 μm), filiform to cylindrical, straight or slightly curved to sigmoid, hyaline, 7-septate, septa often unevenly distributed, not constricted at the septa, asymmetrical, broadly rounded at the apical end and tapering towards the narrowly rounded basal end, with one or two guttules in each cell, 2–3-seriate, rarely 4-seriate, partially overlapping, with a negative or weak dextrinoid reaction in Melzer’s reagent. Anamorph: Unknown.

**Figure 9.**
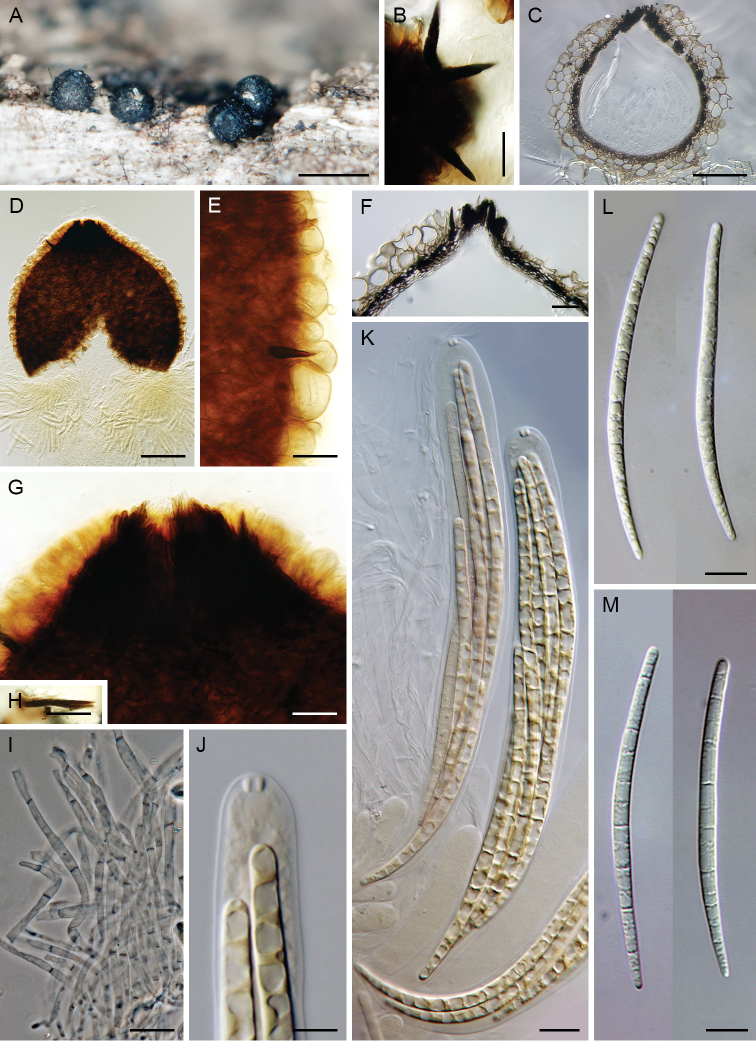
*Paragaeumannomyces sabinianus*. **A** ascomata. **B** ascomatal setae **C** vertical section of ascomal wall **D, E** ascomal wall **F, G** upper part of the ascoma with ostiole surrounded by setae **H** setae from the ostiolar region **I** paraphyses **J** ascal apex with apical ring **K** asci **L, M** ascospores. Images: ILLS00121384 (**A, B, D, E, G–K**); S.M.H. 3824 (**C, F, L**); S.M.H. 3807 (**M**). Scale bars: 500 μm (**A**); 20 μm (**B, E, G, H**); 100 μm (**C, D**); 25 μm (**F**); 5 μm (**J**); 10 μm (**I, K–M**).

##### Other specimen examined.

USA – North Carolina • Macon Co., Coweeta Hydrological Laboratory; 27 Jun. 1998, on decorticated wood; F.A. Fernández leg.; S.M.H. 3807. • *Ibid.*, Horse Cove Drive & Bull Pen Road, alt. 1000 m; 27 Jun. 1998; on decorticated wood; F.A. Fernández leg.; S.M.H. 3824.

##### Habitat and distribution.

A saprobe on decaying wood, so far known from North America in the USA (North Carolina, Tennessee) (Ellis 1887; Huhndorf and Fernández 2005).

##### Notes.

Huhndorf and Fernández (2005) reported *P. longisporus* (as *Ch. ellisii*) from numerous collections from North America; the phylogenetic analysis of ITS sequences of six specimens resolved this species as a statistically unsupported clade with two strongly supported subclades. Although Huhndorf and Fernández (2005) described *P. longisporus* with setae scattered over the entire ascoma, in discussion, they admitted the presence of setae also around the ostiole: “In *C. ellisii*, *C. raciborskii* and *C. panamensis* the setae tend to be scattered over the entire surface of the ascomata, however some specimens of *C. ellisii* may have setae concentrated only at the apex.”

Barr (1993) described the ostiole of the holotype of *S. longispora* surrounded by a crown of dark brown, stiff setae. We examined three collections tentatively identified as *P. longisporus* from North America (ILLS00121384, ILLS00121385, ILLS00121386) and in each the ostiole was delimited by densely aggregated acute setae. Apart from the ostiolar setae, additional setae were scattered over the entire ascoma, but they differed by their density among collections. The ascomata and asci of these three collections are comparable in size; the main difference lies in the ascospore length. The specimen ILLS00121384 has longer [(64.5–)68.5–86.5(–88.5) μm] ascospores compared to ILLS00121385 and ILLS00121386, which have shorter [(50.5–)52.5–68 μm] ascospores corresponding to the size given by Barr (1993) for the *S. longispora* holotype. In the description of *P. longisporusfide*Huhndorf and Fernández (2005), a wide range of ascospore lengths [(40–)50–75(–80) μm] is given, the upper limit matching the ascospore size of ILLS00121384.

In our ITS-28S phylogeny, *P. longisporusfide*Huhndorf and Fernández (2005) was resolved as a strongly supported clade (100/1.0/100) with two subclades. The first subclade (100/1.0/100) was introduced as a new species *P. sabinianus*, including ILLS00121384 (Fig. 9), S.M.H. 3807 (Huhndorf and Fernández 2005: fig. 13) and S.M.H. 3824 (Huhndorf and Fernández 2005: fig. 15) with longer ascospores, distinguished from the second subclade *P. longisporus* (99/1.0/100) with shorter ascospores (Fig. 8). The anamorph of *P. sabinianus* is unknown; our specimen was not isolated in axenic culture and the strains S.M.H. 3807 and S.M.H. 3824 formed only sterile mycelium in vitro (Huhndorf and Fernández 2005).

#### Paragaeumannomyces smokiensis

Taxon classificationFungiChaetosphaerialesChaetosphaeriaceae

Réblová & A.N. Mill. sp. nov.

84D781EB-FF49-580A-89E0-73939F49C4D0

836539

Figure 10

##### Typification.

USA – Tennessee • Sevier Co., Great Smoky Mountains National Park, Greenbrier, alternative side trail to Whaley Cemetery; alt. 549 m; 10 Jul. 2005; on decaying wood; A.N. Miller & A.M. Stchigel leg.; A.N.M. 466 (**holotype**: ILLS00121398!).

##### Etymology.

Named after the Great Smoky Mountains National Park from where it was collected.

##### Description on the natural substrate.

Teleomorph: Ascomata perithecial, non-stromatic, superficial, solitary or in small groups, 270–390 μm diam, 320–400 μm high, subglobose to broadly conical, finely roughened, dark reddish-brown, glabrous except for the black papilla with dark brown, stiff, acute setae, 9.5–13.5 × 2–2.5 μm, densely aggregated around the ostiole. Ostiole periphysate. Ascomatal wall leathery, three-layered; outer layer of textura angularis consisting of globose to subglobose, reddish-brown cells; middle layer composed of brick-like, brown cells; inner layer of flattened, thin-walled, subhyaline cells. Paraphyses abundant, hyaline, longer than the asci, tapering. Asci (134–)140–174(–189) × 11–13(–14) μm (mean ± SD = 158.2 ± 19.4 × 12.3 ± 0.9 μm), cylindrical-fusiform, stipitate, apically narrowly rounded, ascal apex non-amyloid with a distinct apical annulus 2–2.5 μm wide, 1–1.5 μm high. Ascospores (58–)60.5–80.5 × (3–)3.5–4.5(–5) μm (mean ± SD = 69.8 ± 6.3 × 4.0 ± 0.5 μm), filiform to cylindrical, straight or slightly curved, hyaline, 9–11-septate, septa often unevenly distributed, not constricted at the septa, asymmetrical, rounded at the apical end, tapering towards the basal end, with one or two guttules in each cell, 2–3-seriate or 4-seriate and partially overlapping, seldom in a single fascicle. Anamorph: Unknown.

**Figure 10.**
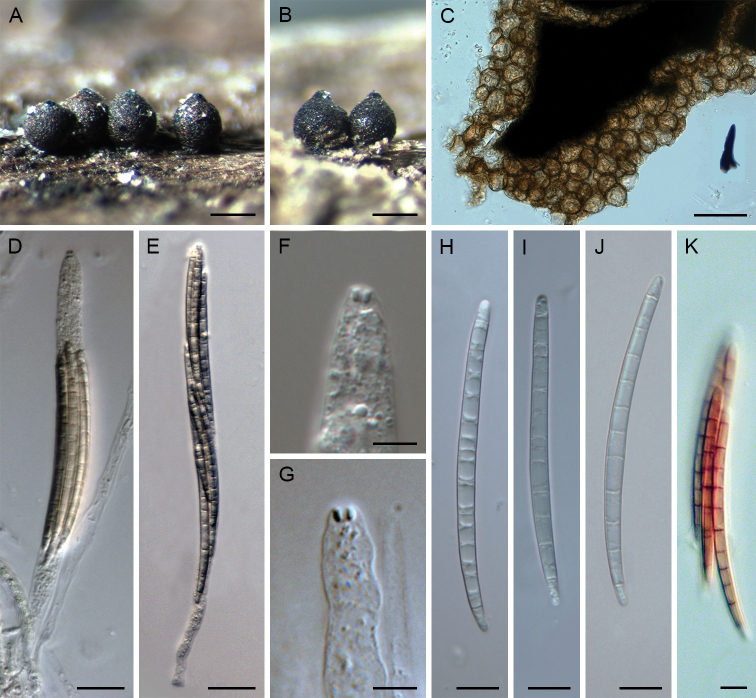
*Paragaeumannomyces smokiensis* (ILLS00121398). **A, B** ascomata **C** globose cells of the outer layer of the ascomal wall and an ostiolar seta **D, E** asci **F, G** ascal apex with apical annulus **H–K** ascospores. Scale bars: 250 μm (**A, B**); 50 μm (**C**); 20 μm (**D, E**); 10 μm (**F–K**).

##### Habitat and distribution.

A saprobe on decaying wood, known only from the USA.

##### Notes.

The present species is most similar to *P. abietinus*, the only member of the genus known from Europe. They share dark reddish-brown, glabrous ascomata with short setae surrounding the ostiole. Although the size of ascospores of both species overlap, *P. abietinus* differs from *P. smokiensis* by longer [(185–)195–240 × 12–14.5(–15.5) μm] asci and slightly longer and wider ascospores [(62–)65–87 × (3.5–)4–5.5 μm] with usually less septa [(5–)7–9(–11)].

#### Paragaeumannomyces sphaerocellularis

Taxon classificationFungiChaetosphaerialesChaetosphaeriaceae

Matsush., Matsush. Mycol. Mem. 10: 156. (2003) [2001].

893CEAFD-B654-5BC5-A916-AD8E431B547F

##### Habitat and distribution.

The species was described from dead twigs of an unknown broadleaf tree and is so far known only from the subtropical climate zone of the northern hemisphere in Japan, Wakayama Prefecture (Matsushima 2003).

##### Notes.

*Paragaeumannomyces sphaerocellularis* is similar to *P. panamensis* (Huhndorf and Fernández 2005; Perera et al. 2016) in the morphology of reddish-brown, setose ascomata with acute, dark, opaque setae scattered over the entire surface and hyaline ascospores, but differs from it in larger ascomata 200–350 × 300–425 μm vs 185–235 × 190–270 μm, slightly shorter asci 105–125 μm vs 123–140 µm, and 5–10-septate ascospores longer in their upper range 65–90 × 3–4 μm vs always 7-septate, shorter ascospores 65–75 × 3–4 of *P. panamensis*. Although the size of the asci may vary, often dependent on the arrangement of ascospores, shorter ascospores with the constant occurrence of seven septa of *P. panamensis* is considered an important character. In other *Paragaeumannomyces* species with 7-septate ascospores, such as *P. elegans, P. lapazianus* and *P. rubicundus*, the number of seven septa remains constant and is considered a diagnostic feature. While *P. sphaerocellularis* was collected only once in Japan, two collections of *P. panamensis* originating from Panama and Thailand suggest that this species has a pantropical distribution. Although the two species are remarkably similar, without molecular evidence we prefer to consider them as separate. For a detailed comparison, see the key.

#### Striatosphaeria castanea

Taxon classificationFungiChaetosphaerialesChaetosphaeriaceae

Réblová & J. Fourn. sp. nov.

E1BF3D38-8FE2-5CFB-941E-87B1ECD83ABF

836540

Figure 11

##### Typification.

French Guiana • Maripasoula, Saül, sentier des gros arbres, disturbed secondary rainforest; alt. 200 m; 25 Aug. 2018; on the bark of decaying woody liana on the ground associated with *Xylaria papillatoides*; C. Lechat leg.; GY.J.F. 18140-1 (**holotype**: PRA-16328!, ex-type culture CBS 145352).

##### Etymology.

*Castanea* (Latin) chestnut-coloured, referring to the colour of conidia.

##### Description on the natural substrate.

Teleomorph: Ascomata perithecial, non-stromatic or formed on rudimentary basal stroma, superficial, solitary or in small groups or dense clusters, 160–200 μm diam, 170–220 μm high, subglobose to broadly conical, dark brown, glabrous, papillate. Ostiole periphysate. Ascomatal wall fragile, carbonaceous, 20–27 μm thick, two-layered. Outer layer of textura prismatica, consisting of brown, polyhedral cells with opaque walls. Inner layer of textura prismatica, consisting of several rows of thin-walled, hyaline, flattened cells. Paraphyses sparse, partially disintegrating at maturity, septate, 3–5 μm wide, tapering to ca. 2.5 μm, longer than the asci. Asci (75–)78–97(–102) × (10.5–)11–14.5 μm (mean ± SD = 87.3 ± 5.0 × 12.5 ± 1.1 µm), (56.5–)65–77(–82.5) μm (mean ± SD = 70.0 ± 4.9 µm) long in the sporiferous part, cylindrical-fusiform, stipitate, apically obtuse, ascal apex with a shallow, non-amyloid apical annulus 3.5–4.5 μm wide, 1–1.5 μm high. Ascospores (10.5–)11–13.5(–14.5) × (5.5–)6–7.5 μm (mean ± SD = 12.2 ± 0.5 × 6.7 ± 0.5 µm), ellipsoidal-fusiform, dark brown to chestnut brown, 1-septate; septum median, dark brown, with a central pore, not constricted or slightly constricted at the septum, with longitudinally arranged darker brown ridges alternating with lighter brown furrows, ascospores uniseriate or obliquely uniseriate in the ascus. Anamorph: Codinaea-like, not present on the nature substrate.

**Figure 11.**
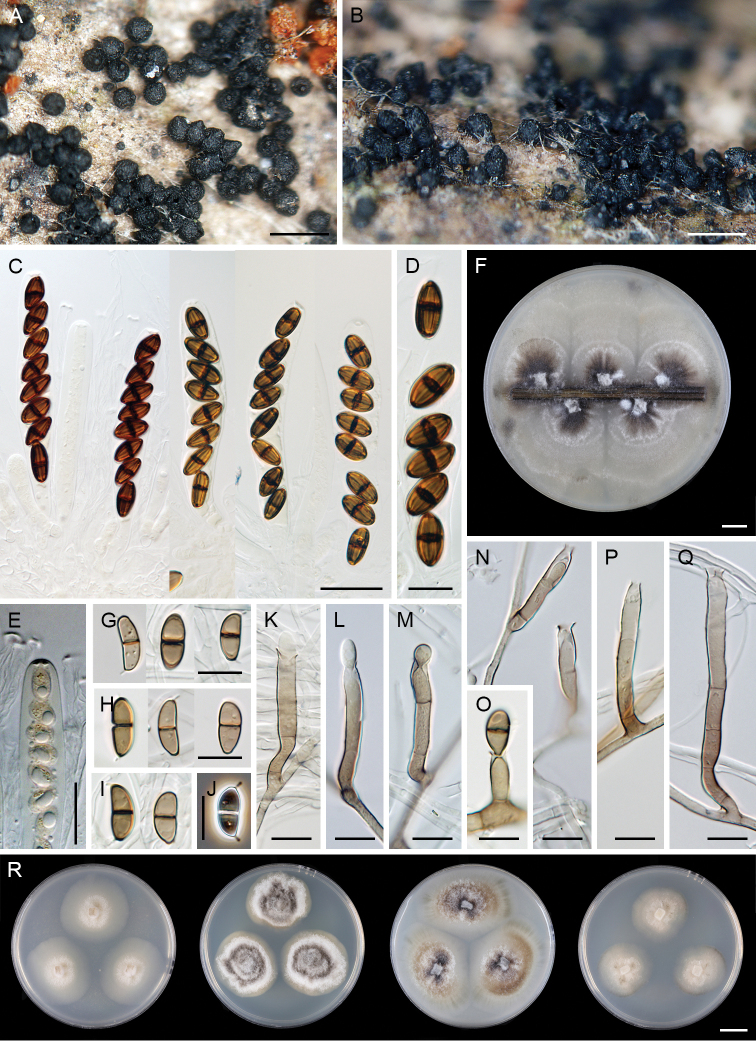
*Striatosphaeria castanea* (CBS 145352). **A, B** ascomata **C** asci **D** ascospores **E** ascal apex with apical annulus **F** colonies on CMA with an *Urtica dioica* stem after 8 wk **G–J** conidia **K–Q** conidiophores **R** colonies on CMD, MLA, OA and PCA after 4 wk (from left to right). Scale bars: 500 μm (**A, B**); 20 μm (**C, E**); 10 μm (**D, G–Q**); 1 cm (**F, R**).

##### Description on CMA with sterile stems of *Urtica dioica*.

Colonies effuse, vegetative hyphae hyaline, branched, 2.5–3.5 μm wide. Conidiophores macronematous, mononematous, 22–66 μm long, 3.5–5 μm wide near the base, erect, straight, cylindrical, several-septate, brown, paler towards the apex, unbranched, smooth-walled, or reduced to single conidiogenous cells. Conidiogenous cells (9.5–)12.5–25(–33) × 4–4.5 μm, tapering to 2.5–3.5 μm just below the collarette, monophialidic, integrated, terminal, cylindrical to cylindrical-lageniform, pale brown, subhyaline towards the apex, smooth-walled; collarettes funnel-shaped, 4–6.5 μm wide, (1.5–)2–2.5 μm deep. Conidia (10–)11–13.5 × 4–5.5 μm (mean ± SD = 11.9 ± 0.8 × 4.8 ± 0.4 µm), reniform to ellipsoidal, straight or slightly curved, asymmetrical, narrowly rounded at the apical end, truncate at the basal end, brown, 1-septate, not constricted or slightly constricted at the septum, with 1–2.5 μm long, hyaline setulae at each end, smooth-walled, in slimy droplets, dark brown in mass.

##### Culture characteristics.

On CMD colonies 23–25 mm diam, circular, flat, margin entire, lanose, floccose, funiculose at the centre, cobwebby towards the periphery, whitish with irregular pale brown spots due to pigmented funiculose mycelium, with an isabelline outer zone of submerged growth; reverse beige. On MLA colonies 22–25 mm diam, circular, raised, margin entire, lanose, floccose, zonate, with grey, brown and white zones, with an isabelline outer zone of submerged growth; reverse dark grey. On OA colonies 31–33 mm diam, circular, flat, margin entire, sparsely lanose, floccose, cobwebby at the margin, zonate, whitish, colony centre with irregular dark brown spots due to pigmented submerged mycelium, pale brown towards the margin, with an olivaceous outer zone of submerged growth, dark brown pigment diffusing to agar at the colony centre; reverse olivaceous grey. On PCA colonies 17–19 mm diam, circular, slightly convex centrally, margin entire, lanose, floccose becoming cobwebby towards the periphery, isabelline to light beige with irregular brown spots due to pigmented mycelium; reverse light beige. Sporulation abundant on CMD, CMA with *Urtica* stems and PCA, sparse on MLA and OA.

##### Other specimen.

Brazil • Bahia; isolated from roots of *Encyclia ghillanyi*; isolate monte6.2; GenBank (ITS): KC928368, unpublished. (Specimen not available).

##### Habitat and distribution.

*Striatosphaeria castanea* occurs on the bark of woody liana and as an endophyte of *Encyclia ghillanyi*. It is known from South America in Brazil and French Guiana.

##### Notes.

*Striatosphaeria codinaeophora* closely resembles *S. castanea*, but differs in having larger [(130–)140–160(–170) × 25–35(– 40) μm] asci, [(17–)19–23(–26) × (6–)7–9(–10) μm] ascospores and (15–20 × 4.5–6 μm) conidia (Samuels and Müller 1978). Based on the present phylogeny (Fig. 1) and comparison of ITS sequences, *S. castanea* has also been recorded as an endophyte, isolated from roots of *Encyclia ghillanyi*, a rupiculous orchid inhabiting rock surfaces in semiarid areas in the Bahia state of northern Brazil (strain monte6.2, ITS: KC928368, Almeida et al. unpublished).

#### Dendrophoma cytisporoides

Taxon classificationFungiChaetosphaerialesChaetosphaeriaceae

Sacc., Michelia 2: 4. 1880.

5DE41B6C-6613-5499-A8FF-47B85A62A63E

Figure 12

≡ Phoma cytisporoides Sacc., Michelia 1: 522. 1879.
≡ Phoma cytisporoides subsp. punicina Sacc., Michelia 2: 273. 1881.
≡ Dendrophoma cytisporoides var. punicina (Sacc.) Sacc., Syll. fung. 3: 180. 1884.
≡ Dendrophoma cytisporoides var. pruni-virginianae Sacc., Riv. Accad. di Padova 33: 169. 1917.
= Dendrophoma punicina (Sacc.) Sacc., Rabenh. Krypt.-Fl., Edn. 2, 1(6): 409. 1901.

##### Description on the natural substrate.

Teleomorph: Ascomata perithecial, non-stromatic, immersed becoming erumpent, in small groups or dense caespitose clusters on the bark of the host, 120–150 μm diam, 180–200 μm high, subglobose to broadly conical, dark brown, glabrous, papillate. Ostiole periphysate. Ascomatal wall fragile, carbonaceous, 22–28 μm thick, two-layered. Outer layer of textura prismatica, consisting of brown, polyhedral cells with opaque walls. Inner layer of textura prismatica, consisting of several rows of thin-walled, hyaline, flattened cells. Paraphyses sparse, persistent, septate, anastomosing, 2–2.5 μm wide, tapering to ca. 1–1.5 μm, longer than the asci. Asci (66–)69–88.5(–92) × 6.5–8 μm (mean ± SD = 77.5 ± 5.9 × 7.3 ± 0.5 µm), (35.5–)43.5–63(–69) μm (mean ± SD = 53.9 ± 5.7 µm) long in the sporiferous part, unitunicate, arising from densely branched, short ascogenous hyphae, 8-spored, cylindrical to cylindrical-clavate, stipitate, apically broadly rounded, ascal apex with an indistinct, non-amyloid apical annulus visible only with the PC illumination, ca. 1.5 μm wide, 1.5–2 μm high. Ascospores 8–10.5(–11) × 2.5–3.5 μm (mean ± SD = 9.5 ± 0.6 × 3.0 ± 0.3 µm), fusiform, hyaline, 1-septate, not constricted at the median septum, uniseriate, obliquely uniseriate or partially biseriate in the ascus. Anamorph: Not observed.

**Figure 12.**
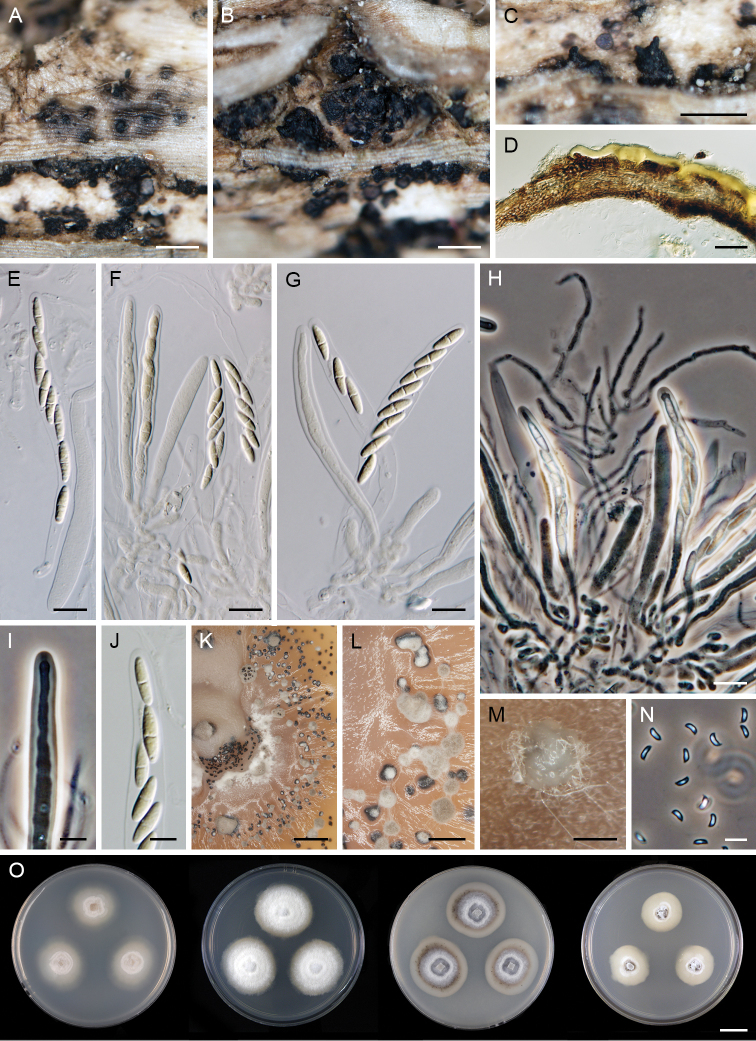
*Dendrophoma cytisporoides* (CBS 144107). **A–C** ascomata **D** vertical section of the ascomal wall with remnants of the periderm **E–G** asci **H** paraphyses, asci and ascogenous hyphae **I, J** ascal apex **K, L** sterile primordia of sporodochia on MLA after 6 mo **M** sporodochium on OA after 10 wk **N** conidia **O** colonies on CMD, MLA, OA and PCA after 4 wk (from left to right). Scale bars: 300 μm (**A–C, K**); 20 μm (**D**); 10 μm (**E–H**); 5 μm (**I, J, N**); 100 μm (**L, M**); 1 cm (**O**).

##### Description on OA.

Colonies effuse, vegetative hyphae hyaline, 2–3 μm wide. Conidiomata stromatic, globose becoming cupulate, up to 350 μm diam. Setae absent. Conidiophores macronematous, septate, branched or unbranched, up to 60 μm long, hyaline. Conidiogenous cells 7–13 × 1.5 μm, monophialidic, integrated and terminal or discrete and lateral, subcylindrical, single or in terminal whorls, hyaline, tapering to ca. 1 μm; collarettes indistinct. Conidia 2.5–3.5 × 1–1.5 (mean ± SD = 3.1 ± 0.3 × 1.2 ± 0.1 µm), naviculate to botuliform, with 0.5–1 μm long setulae at each end, aseptate, smooth-walled, in slimy droplets, hyaline in mass.

##### Culture characteristics.

On CMD colonies 18–20 mm diam, circular, flat, margin entire, velvety-lanose, mucoid at the margin, white, isabelline towards the periphery, reverse white. On MLA colonies 18–21 mm diam, slightly convex, circular, margin entire to fimbriate, lanose, floccose, cobwebby at the margin, white, with an isabelline outer zone of submerged growth, reverse isabelline. On OA colonies 19–21 mm diam, circular, flat, raised margin, margin entire, velvety becoming lanose towards the margin, mucoid at the margin, zonate, colony centre grey to whitish-grey with intermediate white zone becoming brown, beige at the margin, reverse grey. On PCA colonies 13–15 mm diam, circular, flat, slightly convex centrally, margin entire, velvety-lanose, floccose becoming mucoid towards the periphery, white, pale brown centrally, with an isabelline outer zone of submerged growth, reverse isabelline. Sporulation absent on all media after 4 wk; abundant on OA and PCA after 10 wk or 4 mo, respectively, on MLA sterile primordia of conidiomata were formed.

**Specimen examined.** Germany • 16 Jan. 2012; on bark and wood of a dead twig of *Buxus sempervivens*; R. Schumacher leg.; (PRA-16322, culture CBS 144107 = IMI 506817).

##### Habitat and distribution.

Saprobic on decaying wood and bark of *Buxus sempervivens*, *Deutzia scabra*, *Rhododendron* sp., and *Ulmus* sp. The species is known from Europe in France, Germany and the Netherlands (Saccardo 1879; Sutton 1965; Crous et al. 2012; this study).

##### Notes.

Our strain sporulated in vitro only after prolonged incubation. On OA it formed globose to pulvinate fertile conidiomata, while on PCA the conidiomata often became confluent. The comparison of ITS and 28S sequences of our strain with those of the epitype strain of *D. cytisporoides*CBS 223.95 (Crous et al. 2012) confirmed they are conspecific; the two strains share 100 % sequence similarity.

## Discussion

Based on morphology, cultivation studies and phylogenetic analysis of the combined ITS-28S loci, seven new species and eight new combinations are introduced in the Chaetosphaeriaceae. *Paragaeumannomyces* (Matsushima 2003) is proposed for the monophyletic, strongly supported clade of former *Chaetosphaeria* species (Figs 1, 2) characterised by scolecosporous, multiseptate, asymmetrical, hyaline to light pink ascospores and unique three-layered ascomatal wall. The unusual wall was first brought to attention by Carroll and Munk (1964). The colour of the outer wall ranges from white, whitish-yellow, ginger-brown to reddish-brown, russet to dark brown and is composed of thin-walled cells of textura angularis, which may contain pale purple pigment when fresh, occasionally with red surface crystals. Setae, if present, are scattered over the entire ascoma or only surround the ostiole. They are stiff, acute, dark brown with opaque walls, arise from the middle, carbonaceous layer and penetrate the outer layer of globose cells. Members of the genus occur on decayed plant material, especially on strongly decayed decorticated wood.

Examination of our material of *Paragaeumannomyces* revealed that ascospores of four species exhibit a strong dextrinoid reaction in Melzer’s reagent, namely *P. abietinus*, *P. albidus*, *P. granulatus* and *P. smokiensis*. The ascospores turned reddish-brown except for the end cells, which remained partially hyaline, especially at the tips. Interestingly, these species share glabrous, non-setose ascomata or only minute setae are arranged around the ostiole. The chemical reaction is visible in ascospores without guttules, which otherwise fill the cells and obscure the colour. The ascospores of *P. elegans*, *P. longisporus* and *P. sabinianus* exhibit a negative or weak dextrinoid reaction; some mature ascospores turned light pink-brown. These species share setose ascomata, sometimes with ostioles surrounded by minute setae. Although more species need to be examined to evaluate this character, we hope it is not premature to argue that the dextrinoid reaction of ascospores is species-specific and has the potential to become another diagnostic feature facilitating species identification. Because we did not examine all known species of *Paragaeumannomyces*, this character has not been used in the key, but it is mentioned in the species descriptions, if known.

*Paragaeumannomyces*, typified by *P. sphaerocellularis*, encompasses 18 species. The present phylogenetic tree (Fig. 2) contains 12 of them and four subclades labelled *Paragaeumannomyces* sp. 1–4, which represent separate, yet undescribed taxa at the species rank. The molecular data of *P. raciborskii* and *P. sphaerocellularis* are unavailable. The closest relatives to *Paragaeumannomyces* are species of *Exserticlava* and *Chaetosphaeria lignomollis* with a kylindria-like anamorph, characterised by septate, versicolorous or hyaline ascospores, respectively (Fig. 1).

Members of *Paragaeumannomyces* are not easily cultivated and only seldom sporulate in vitro. Huhndorf and Fernández (2005) noted that even isolates from the same specimen varied in their ability to produce the anamorph in culture. The craspedodidymum-like anamorph with usually semi-macronematous to micronematous conidiophores, inflated phialides, deeply flared, cup-shaped collarettes and hyaline non-septate conidia, with or without setulae, was reported for *P. lapazianus*, *P. longisporus*, *P. panamensis*, *P. rubicundus*, and *Paragaeumannomyces* sp. 1–4, while the chloridium-like synanamorph is known only in *P. longisporus* and *Paragaeumannomyces* sp. 2 (Huhndorf and Fernández 2005; Perera et al. 2016). The systematic placement of *Craspedodidymum* (Holubová-Jechová 1972), typified by *C. elatum*, is unknown. The genus was erected for a hyphomycete forming effuse colonies on an old petiole of *Phoenix canariensis* in a green house in the Czech Republic. To date, 15 binomials were introduced in the genus (Index Fungorum). *Craspedodidymum elatum* differs from *Paragaeumannomyces* anamorphs by macronematous, dichotomously branched conidiophores and non-septate, dark brown conidia with a basal hilum. In hyaline, unicellular, globose or triangular conidia with setulae, the *Paragaeumannomyces* anamorphs also resemble *Bahusutrabeeja* (Subramanian and Bhat 1977) and *Nawawia* (Marvanová1980), respectively. *Bahusutrabeeja* and *Nawawia* are similar to each other but differ in shape of conidia. *Bahusutrabeeja*, typified with *B. dwaya*, forms globose conidia on solitary conidiophores, while *Nawawia*, based on *N. filiformis*, have conidia triangular, round-tetrahedral or obpyramidal-shaped on conidiophores arising from small stromata. Their molecular data (Yang et al. 2016; Vu et al. 2019) suggest a distant relationship to *Paragaeumannomyces*.

The original *P. longisporus* clade with two strongly supported subclades was recognized as two species, the short-spored *P. longisporus* and the long-spored *P. sabinianus*. In general, a high degree of ITS sequence variability and more or less uniform teleomorphic phenotype pose special problems in species identification, especially in *P. raciborskii*, which was resolved as polyphyletic (Huhndorf and Fernández 2005; this study). Morphology of this species was studied by Huhndorf and Fernández (2005) based on more than 100 species, mostly from the neotropics. The broad species concept of *P. raciborskiifide*Huhndorf and Fernández (2005) includes specimens with wider ascomata, longer ascospores with less septa and longer asci than reported in the protologue (Penzig and Saccardo 1897) and re-description of this species prepared by Carroll and Munk (1964); for details see above. Although no significant variability among ascomata, asci and ascospores was encountered, Huhndorf and Fernández (2005) reported intraspecific variability regarding setae, which were lacking in some specimens. On the other hand, certain variability at the anamorphic level, typical of many natural groups of the Chaetosphaeriaceae, also occurs in *P. raciborskiifide*Huhndorf and Fernández (2005) and to some extent delimits the four subclades. One isolate (S.M.H. 3119) produced a craspedodidymum-like anamorph with triangular conidia with setulae, while others with the morphologically similar anamorph produced globose, non-setulate conidia and can be further distinguished by formation of the chloridium-like synanamorph (S.M.H. 2036, S.M.H. 2132), or its absence but are separated by dark, purplish-brown phialides (S.M.H. 2017) or light brown phialides (S.M.H. 3014) (Huhndorf and Fernández 2005). A close comparison of the descriptions of the holotype of *P. raciborskii* (Penzig and Saccardo 1897; Carroll and Munk 1964) with collections gathered by Huhndorf and Fernández (2005) and identified as *P. raciborskii* confirms that none of the four subclades inferred in the ITS-28SML tree (Fig. 2) could be delimited as *P. raciborskii* s. str. From a biogeographical perspective it is more likely that they belong to different species entirely. Therefore, the name *P. raciborskii* for these strains was rejected in our phylogeny; instead, the four subclades were designated *Paragaeumannomyces* sp. 1–4.

*Codinaea* (Maire 1937) is one of the largest genera of the Chaetosphaeriaceae with a turbulent taxonomic history. Based on a cluster analysis of phialidic dematiaceous hyphomycetes, Arambarri and Cabello (1989) considered *Codinaea*, with usually falcate, septate or non-septate conidia bearing setulae at both end, and *Dictyochaeta* (Spegazzini 1923) with non-setulate, non-septate, cylindrical and asymmetrical conidia, congeneric. Since then, *Dictyochaeta* (syn. *Codinaea*) became a broadly circumscribed genus with more than 100 species and the new morphological concept was followed by many mycologists. Réblová and Seifert (2007) confirmed with DNA sequence data that *Dictyochaeta fuegiana*, the type species of the genus, is a member of the Chaetosphaeriaceae. Based on the ITS-28S phylogeny (Fig. 1), *D. fuegiana* is unrelated to morphologically similar species with setulate conidia, classified in *Codinaea* or *Dictyochaeta*, and resolved as polyphyletic, which is in agreement with Lin et al. (2019) and Réblová et al. (2020). However, in the absence of molecular DNA data of *Codinaea aristata*, the generic type, it is difficult to delimit *Codinaea* phylogenetically. The morphological traits delimiting the new species *C. paniculata*, i.e. presence of setae, unbranched and shorter conidiophores growing at the base of the setae with monophialidic conidiogenous cells in vivo and falcate, non-septate conidia with setulae at both ends, best match those of *Codinaea*. In the subclade where *C. paniculata* was clustered, several species sharing the same *Codinaea* morphotype were present, namely *C. assamica* (Hughes and Kendrick 1968), *D. siamensis* (Liu et al. 2016) and *D. terminalis* (Lin et al. 2019).

Based on published records, *Striatosphaeria* is an uncommon lignicolous genus with a known distribution in the neotropics. It was introduced by Samuels and Müller (1978) based on two Brazilian collections of *S. codinaeophora*. Additional specimens of *S. codinaeophora* known to us were collected on decaying wood of *Dacryodes excelsa* and *Nectandra turbacensis* and unidentified hosts in Costa Rica, French Guiana and Puerto Rico (Réblová and Winka 2000; Fernández et al. 2006; S.M. Huhndorf pers. data). The codinaea-like anamorph develops only in axenic culture. The conidia are asymmetrical, brown, 1-septate with minute, hyaline setulae at each end. Although *S. codinaeophora* was described with non-setulate conidia (Samuels and Müller 1978), a photograph of conidia with setulae accompanied the *S. codinaeophora* lineage on a phylogenetic tree (Fernández et al. 2006: fig. 1, 6g). The setulate conidia were also present in the new species, *S. castanea*. It is probable that setulae are formed later, after conidia detach from the conidiogenous cells. The conidia with setulae at both ends are formally introduced in *Striatosphaeria* for the first time in this study.

The genus *Dendrophoma* with a single species, *D. cytisporoides*, was proposed by Saccardo (1880) for fungi with phoma-like fruit bodies, botuliform hyaline conidia and conidiogenous cells arranged in a verticillate fashion. Sutton (1965) lectotypified *Phoma cytisporoides* (Saccardo 1879) and reported additional characters not mentioned by Saccardo in the protologue, i.e. dark brown, acute setae accompanying conidiomata and minute, unbranched setulae at both ends of conidia. Sutton (1965) compared *D. cytisporoides* with *Dinemasporium graminum*, the type species of the genus, and reduced *Dendrophoma* to synonymy with *Dinemasporium* (Léveillé 1846). Using nuclear ribosomal loci, Crous et al. (2012) re-established *Dendrophoma* and placed the genus in the Chaetosphaeriaceae, where it emerged as a separate lineage from *Dinemasporium*. The anamorph-teleomorph relationship of *Dendrophoma* has been established for the first time in this study. The teleomorph is morphologically similar to *Chaetosphaeria* (Tulasne and Tulasne 1863), but differs in having immersed to erumpent ascomata, densely branched ascogenous hyphae (Fig. 12H), the ascal apex lacking a visible discharge mechanism, which can only be seen as a minute apical ring with PC illumination (Fig. 12I) and a morphologically distinct anamorph forming stromatic, stipitate, cupulate sporodochial conidiomata.

## Supplementary Material

XML Treatment for Codinaea paniculata

XML Treatment for Paragaeumannomyces

XML Treatment for Paragaeumannomyces abietinus

XML Treatment for Paragaeumannomyces albidus

XML Treatment for Paragaeumannomyces bombycinus

XML Treatment for Paragaeumannomyces elegans

XML Treatment for Paragaeumannomyces garethjonesii

XML Treatment for Paragaeumannomyces granulatus

XML Treatment for Paragaeumannomyces lapazianus

XML Treatment for Paragaeumannomyces longisporus

XML Treatment for Paragaeumannomyces panamensis

XML Treatment for Paragaeumannomyces raciborskii

XML Treatment for Paragaeumannomyces rubicundus

XML Treatment for Paragaeumannomyces sabinianus

XML Treatment for Paragaeumannomyces smokiensis

XML Treatment for Paragaeumannomyces sphaerocellularis

XML Treatment for Striatosphaeria castanea

XML Treatment for Dendrophoma cytisporoides
